# Employees’ preferences on organisational aspects of psychotherapeutic consultation at work by occupational area, company size, requirement levels and supervisor function – a cross-sectional study in Germany

**DOI:** 10.1186/s12889-023-15255-0

**Published:** 2023-02-16

**Authors:** Fiona Kohl, Peter Angerer, Jeannette Weber

**Affiliations:** Institute of Occupational, Social and Environmental Medicine, Centre for Health and Society, Medical Faculty, Heinrich-Heine-University Düsseldorf, Moorenstraße 5, 40225 Düsseldorf, Germany

**Keywords:** Mental health, Workplace, Occupational health, Psychotherapy, Patient preference, Psychotherapeutic care

## Abstract

**Background:**

Common mental disorders affect a significant proportion of the population worldwide at any given time. Psychotherapeutic consultation at work offers employees with mental distress short-term and low-threshold access to psychotherapeutic treatment. However, this offer is only accepted by one to two percent of the employees to whom it is offered. Taking into account employees ‘ preferences regarding organisational aspects might increase the use of psychotherapeutic consultation at work. This study therefore aimed to identify preferences on organisational aspects of psychotherapeutic consultation at work among employees of diverse occupational areas, company sizes, supervisor functions and job requirement levels.

**Methods:**

A total of 755 employees were recruited via advertisements on social media (Instagram, Facebook and LinkedIn). Participants rated on a 5-point Likert scale their agreement to different implementation options of psychotherapeutic consultation at work: type (in-person/video/telephone), location (on/outside company premises), time (within/outside working hours), scope (diagnostic/diagnostic + treatment) and purpose (private/occupational). Additionally, the maximum accepted distance to the location of consultation was assessed. Various analyses of variances (ANOVA) were conducted to determine differences in agreement to implementation options within each organisational aspect and to analyse differences between occupational areas, company sizes, requirement levels and between employees with and without supervisor function.

**Results:**

Participants indicated a preference for in-person psychotherapeutic consultation that takes places outside company premises and outside working hours. Furthermore, they preferred offers including diagnostic and treatment sessions compared to offers including diagnostic sessions only. Even though participants agreed that consultation should be offered for all purposes, agreement for occupational issues was stronger than for private issues. For some implementation options, the level of agreement varied according to occupational field, company size, supervisor function and level of requirement. However, these differences did not affect the key findings mentioned above.

**Conclusion:**

Those findings give practical indications on the organisational design of psychotherapeutic consultation at work. The results suggest that in-person consultation outside company premises and working hours combining diagnostic and treatment sessions will be accepted by employees regardless of their occupational area, company size, supervisor function and requirement level.

**Supplementary Information:**

The online version contains supplementary material available at 10.1186/s12889-023-15255-0.

## Introduction

Although a large number of people suffer from mental disorders every year [1–4], a large proportion of patients does not receive any treatment (e.g. psychotherapy, primary care) despite being in need [5–7]. Inadequate treatment can lead to high burdens for individuals as well as to high costs for companies and social security systems [5, 8] due to early retirement, long periods of work incapacity and reduced productivity at the workplace [9–12]. Optimizing access to psychotherapeutic care to the needs of individuals with mental disorders is therefore of high public health interest.

Reasons for not seeking appropriate treatment are already widely studied [13–17]. Attitudinal barriers – such as the wish to handle the disease by oneself, low perceived need for treatment, concerns about stigma and the belief that treatment will not help – predominate over structural barriers [13–15]. The main structural barriers reported are the lack of knowledge about where to get help, low availability of mental health services (e.g. long waiting times), financial concerns about treatment costs, language barriers and no available means of transportation to the treatment location [13, 16, 17].

Therefore, approaches that offer low-threshold access to care are recommended [11, 18, 19]. In this context, the workplace receives increasing attention when it comes to early diagnosis and treatment of employees with mental distress. Psychotherapeutic consultation at work is a service in which a company promotes short-term access to psychotherapeutic sessions including diagnosis and eventually treatment for its employees [20–22]. This consultation is provided by a psychological or medical mental health care specialist who is usually not employed by the company. However, the consultation may be financed by the company (as an external contractor) [23]. Anonymity should be guaranteed as far as possible and the employer will not be informed about who has received consultation. The service is voluntary and free of charge for employees. There are similar international concepts usually termed Employee Assistance Program (EAP) [24]. Yet, EAP differs in some aspects, as for example EAP counsellors are not necessarily psychotherapists. Compared to psychotherapeutic consultation at work, EAP also offers short-term counselling but usually no treatment or diagnosis [25].

Results of previous studies show that people who are offered psychotherapeutic consultation at work can be reached at an early stage of disease [26] and may return to work after shorter periods of sickness absence compared to those who remain in standard care [27, 28]. Furthermore, a previous study showed that 90% of employees accepted recommended treatment in usual care after receiving comprehensive psychotherapeutic diagnostic consultation in the context of a workplace intervention [22]. On an economic level, analyses have shown that early diagnosis and treatment of depression at work can lead to significant economic benefits for companies [8]. An ongoing randomised controlled trial is additionally investigating the impact of psychotherapeutic consultation at work on days of sickness absence due to mental illness [21].

Although previous study results are promising, experience from practical implementation shows that only about one to two percent of employees who have access to it actually make use of psychotherapeutic consultation at work [29]. Comparative numbers can be drawn from the already widely implemented EAP [30–32]. While some surveys provide similar numbers of one to two percent, others show rates of up to ten percent [30–32]. However, these numbers still do not reflect the high prevalence of depression and work-related stress at work [33, 34]. It is therefore advisable to evaluate the needs and demands that employees have concerning psychotherapeutic consultation at work in order to increase its acceptance and use. In this context, a qualitative study indicated that organisational aspects such as scope (e.g. number of sessions), purpose (e.g. work-related or private aspects) and location of consultation need to be considered when implementing psychotherapeutic consultation at work within companies [23]. For example, it was argued that psychotherapeutic consultation outside company premises could counteract fear of stigmatisation [23]. Furthermore, while some services only provide diagnostic sessions [22], others also include further treatment sessions [21, 35]. In addition, there are different approaches described as to where (e.g. on or outside company premises) and when (e.g. within or outside working hours) psychotherapeutic consultation should be offered [20]. Since video-based consultations are proven to be an adequate alternative to in-person consultation in psychotherapy [36, 37], it should be assessed whether digital solutions offer an attractive form of psychotherapeutic consultation at work for employees. This latter aspect might especially be relevant to increase coverage in rural areas.

Occupation-specific aspects are likely to play a role in employees’ preferences regarding the above mentioned organisational aspects of psychotherapeutic consultation at work and consequently regarding its acceptance and utilisation [38, 39]. For instance, previous research shows that the prevalence of mental illnesses varies between different occupational areas [40]. In particular, occupations with regular customer contact (e.g. service area) show a high prevalence of depression [40]. At the same time, implementation of health interventions in this occupational area appears particularly difficult, because employees might work at different locations or times [39]. Job requirement levels, defined by the number of years that it takes to achieve professional qualification, might also be related to varying preferences regarding organisational aspects of psychotherapeutic consultation at work [41]. For example, previous study results show that more years of education as well as a higher educational degree are related to the use of psychotherapeutic treatment [42]. Furthermore, company size seems to play a significant role in the implementation of health interventions at work, as small companies are less likely to implement services such as EAP than large companies [38, 43]. Within small companies, lack of financial and human resources are often mentioned as barriers for implementation [43, 44]. In addition, employees of small companies often think that health interventions should not be related to the employer [38]. Nonetheless, previous research indicated that small companies are highly motivated to implement health interventions if they are offered to them free of charge and with a precise implementation guideline [45]. Supervisors are an important facilitator in the implementation of mental health interventions [46]. There are often stigma-related barriers in the workplace, such as the fear of being perceived less competent if a mental illness is disclosed [47, 48]. Actively promoting psychotherapeutic consultation could create an open, health-promoting culture [49]. Therefore, acceptance and support by supervisors also seems very important when implementing a psychotherapeutic consultation at work.

Whether these aspects should actually play a role in implementation of a psychotherapeutic consultation at work is uncertain to date. To the best of our knowledge, no quantitative study has yet explored employees’ preferences regarding the implementation of psychotherapeutic consultation at work taking into account different occupational areas, company sizes, job requirement levels and supervisor function. To increase acceptance and utilisation, these wishes should be taken into account when planning psychotherapeutic consultation in a company. Therefore, this study focuses on the following research questions:

1. Which implementation options do employees prefer regarding the following organisational aspects of psychotherapeutic consultation at work
  - type: in-person/video/telephone
  - location: on/outside company premises
  - time: within/outside working hours
  - scope: diagnostic/diagnostic + treatment
  - purpose of consultation: private/occupational
  - travel distance
2. Do those preferences on implementation options referring to organisational aspects of psychotherapeutic consultation at work differ regarding
  - occupational areas
  - company size
  - job requirement levels
  - supervisor function?

Subgroup analyses will further specifically explore preferences on implementation options of employees with symptoms of depression.

## Methods

### Study design and sample

A cross-sectional design with an online survey was used. Participants were recruited between May and August 2021 via advertisements on the social media platforms Facebook, Instagram and LinkedIn. The following slogan was used in the advertisement: “Can we facilitate access to psychotherapeutic service? With a short questionnaire you can help us to find an answer “. The advertisements were shown to active users who indicated an age between 18 and 65 years and a place of residence in Germany. No other settings were specified, in order to avoid that the advertisement is specifically shown to people with certain interests (e.g. mental health topics).

As recruitment via Facebook and Instagram tends to result in a study population which is overrepresented by female gender and younger age, LinkedIn was additionally used as a recruitment platform [50]. The aim was to reduce the risk for selection bias as well as response bias.

Inclusion criteria were (1) age between 18 and 65 years, (2) current employment contract with at least 15 working hours per week. No other inclusion or exclusion criteria were applied. Data was collected anonymously. Informed consent was obtained in the online questionnaire by ticking a box.

Sample size was calculated with G*Power 3.1 [51] considering a group number of ten occupational areas, a small effect size (f = 0.15) with a power of 80% and two-sided significance levels of 5%. This calculation resulted in a required number of 710 participants. Based on our own experience, we assumed that up to 30% of the participants drop out at an early stage of the questionnaire, so that a sample size of 1,000 persons was aimed at.

In total, our study advertisements reached 843,386 persons. Of these, 2,549 followed the link to the questionnaire and 1,087 agreed to the informed consent form. There were 848 participants remaining after checking compliance of inclusion criteria and 755 participants remaining after checking completeness of data (Facebook & Instagram 505, LinkedIn 15, other 20 (e.g. questionnaire received through friend)). A flow diagram of participant recruitment and selection can be found in Additional file 1.

### Measurements

In total, 42 items with variables regarding implementation options of organisational aspects of psychotherapeutic consultation at work, work-related information, mental health information and sociodemographic characteristics were used for analysis.

#### Preferences for implementation options regarding psychotherapeutic consultation at work

The questionnaire from Reineke [29] consisting of 13 items describing implementation options for five organisational aspects of psychotherapeutic consultation at work was used for this study. An overview on those implementation options is given in Table 1 (see Additional file 2 for the whole questionnaire). The implementation options of this questionnaire were derived from previous qualitative and quantitative research on the implementation of psychotherapeutic consultation at work [20, 35]. The relevance of the questions used for implementation options was confirmed by the results of a recent qualitative study on the implementation of a psychotherapeutic consultation at work [23]. Within this questionnaire, participants were asked about how psychotherapeutic consultation at work should be implemented within their company on a 5-point Likert scale from 1 = no agreement at all to 5 = complete agreement.

**Table 1:** Description of the study questionnaire of implementation options regarding five organisational aspects of consultation at work [29]

Organisational aspect	Implementation options	Number of items
(1) Type of consultation	individual consultation in person, by phone, video-based	3
(2) Location	on company premises, outside company premises	2
(3) Time	within working hours, outside working hours	2
(4) Scope	diagnostic only, diagnostic interview with max. 10 treatment sessions	2
(5) Purpose	occupational burden, private burden, maintain work ability, occupational reintegration	4

Additionally, participants were asked how long they would be willing to travel for consultation starting from home or workplace (< 15 Min, 15 – 30 min., 30 – 45 min., > 45 min., no transport available).

#### Work-related questions

Occupational area and job requirement levels were classified according to the German classification code of 2010 [41]. This classification scheme has two dimensions: In the horizontal dimension, occupations are classified according to their professional expertise. This includes knowledge and skills as well as activities required for an occupation. In this study, the first level of the horizontal dimension consisting of ten occupational areas was used. Participants were thus asked in which of the following occupational areas they are working:

1. Agriculture, forestry, farming and horticulture
2. Production of raw materials and goods and manufacturing
3. Construction, architecture, surveying and technical building services
4. Natural sciences, geography and informatics
5. Traffic, logistics, safety and security
6. Commercial services, trading, sales, hotel business and tourism
7. Business organisation, accounting, law and administration
8. Health care, social sector, teaching and education
9. Philology, literature, humanities, social sciences, economics, media, art, culture and design
10. Military

The vertical dimension of the classification code was used for the categorisation into requirement levels. It describes the degree of complexity of an occupation through four requirement levels: unskilled or semiskilled activities (no vocational qualification or regular one year vocational training), specialist activities (at least two years of vocational training), complex specialist activities (qualification as master craftsman or technician, graduation from a professional academy or university bachelor’s degree), highly complex activities (completed university studies of at least four years) [41]. Company size was defined according to the EU recommendation 2003/361 for micro (1–9 employees), small (10–49 employees) and medium-sized enterprises (50–249 employees; SME) [52]. In addition, two more subcategories for large-sized companies were added (250–999 employees and > 1000 employees). Furthermore, participants were asked about number of working hours (full-time with ≥ 35 h/week/part-time with < 35 h/week), supervisor function (yes/no), remote work (yes/no) and shift work (yes/no).

#### Mental health specific questions

Psychological well-being was measured with the 5-item World Health Organization Well-Being Index (WHO-5). The WHO-5 is a short questionnaire which has been validated as a screening tool for depression and as an outcome variable in clinical trials [53]. Participants were asked about their well-being within the past two weeks on a 6-point Likert scale from 0 = “at no time” to 5 = “all of the time”. An example item is “Within the past two weeks, I have felt calm and relaxed”. A sum score was calculated and multiplied by four resulting in a scale from 0 (worst well-being) to 100 (best well-being) [53]. We used a cut-off score of ≤ 50, which is considered accurate for the screening of depression [53].

In addition, participants were asked whether they had been diagnosed with a mental illness recently or in the past. If they answered “yes”, they were asked if they had received/are receiving treatment for it. The form of treatment (e.g. psychotherapy or primary care) was not specified.

#### Sociodemographic characteristics

Participants were asked about their age (years), gender (male/female/diverse), health insurance (statutory/private) and education categorised according to the International Standard Classification of Education: lower secondary (school leaving certificate after up to 10 years of primary and secondary education), upper secondary (school leaving certificate after 11/12 – 13 years of primary and secondary education with qualification for university entrance) and tertiary education (university degree (including PhD)) [54].

#### Covariates

Gender, age, educational status and the severity of depressive symptoms have been found to be associated with help seeking or different preferences for psychotherapeutic treatment in previous studies and were therefore considered as covariates in this study [42, 55, 56]. Since health insurance can have an influence on mental health care in regular care (e.g. shorter waiting times, scope of treatment sessions) and thus possibly on attitudes towards additional psychotherapeutic offer of psychotherapeutic consultation at work, health insurance was also considered as a control variable [57]. In terms of work-related factors, remote work and shift work were considered, as previous studies showed that the implementation or usage of a psychotherapeutic consultation at work may depend on employees’ work locations and shift systems [58].

### Data analysis

Statistical analyses were performed using R version 4.1.1 (The R Foundation, Vienna, Austria). For descriptive purposes, means and standard deviations were calculated for continuous variables and frequencies and percentages for categorical variables. Repeated-measures analyses of variance (RM-ANOVA) were used to calculate differences in agreement between implementation options within each organisational aspect of psychotherapeutic consultation at work.

For answering the *first research question*, one-way RM-ANOVAs for each of the five organisational aspects (type, location, time, scope, purpose) were calculated for the total study sample. For each model, the organisational aspect with the corresponding implementation options (e.g. type of consultation: in person, telephone, video) was used as a within-subject variable. The accepted distance to the consultation location was presented descriptively for the total sample. Furthermore, subgroup analyses were performed to determine preferences of employees with mental distress. For this purpose, the RM-ANOVAS were repeated in a subgroup of participants with a WHO-5 score of ≤ 50.

For answering the *second research question*, the analyses were repeated using (1) occupational area, (2) company size, (3) requirement level and (4) supervisor function as between-subject variables in separate mixed RM-ANOVAS. This resulted in 20 different mixed RM-ANOVAS: four different RM-ANOVAs for each of the five organisational aspects. Regarding the maximum accepted travel distance, one-way ANOVA analyses were used to determine differences between occupational areas, company size, supervisor function and job requirement level. For these analyses, the answer option "No transport available" was deleted so that the variable could be used on an ordinal scale.

Main and interaction effects were examined for all ANOVA analyses. All statistical analyses were two-tailed using a *p*-value < 0.05 for indicating statistical significance. When an effect was significant, post-hoc analyses were performed. For those analyses, pairwise t-tests for independent samples with Bonferroni correction were calculated to compare agreement to each implementation option of the respective work-related aspect between the characteristics of the work-related variables. Furthermore, pairwise t-tests for dependent samples with Bonferroni correction were calculated to analyse differences in agreement between implementation options for each characteristic of the respective work-related variable.

To control for covariates, repeated measures analyses of covariance (RM-ANCOVA) were conducted considering gender, age, educational status, severity of depressive symptoms (measured by WHO-5), health insurance, remote work and shift work. These variables were first included in one-way RM-ANCOVAs for the total study sample. If significant effects or interactions were found for any of the covariates in at least one of the implementation options, this covariate was also included in subsequent mixed RM-ANCOVAS.

Only participants with complete data for the within-subject and between-subject variables were considered for the analyses.

## Results

### Descriptive results

A comprehensive description of the study population can be found in Table 2. The participants were predominantly female and on average 35 years old. Overall, about half of the participants indicated a current mental health diagnosis. Of those, two-thirds currently received psychotherapeutic treatment. Of those who had received a mental health diagnosis in the past, 91% had received treatment for it. Measured with the WHO-5, 70% of participants were found to have depressive symptoms.

**Table 2:** Description of study population (*n* = 755)

Characteristics	mean (SD)
**Age (years)**	35.37 (± 12.2)
	n (%)
**Gender**	
Female	627 (83)
Male	103 (14)
divers	25 (3)
**Health insurance**	
Statutory (vs. private)	716 (95)
**Education**^a^	
Lower secondary education	130 (17)
Upper secondary education	280 (37)
Tertiary education	345 (46)
**Health specific characteristics**	
Incapacity to work due to mental diagnosis	52 (7)
Incapacity to work due to another illness	14 (2)
Current mental illness (*n* = 728)	350 (48)
Thereof currently in treatment	231 (67)
Mental illness in the past (*n* = 716)	434 (60)
Thereof in treatment in the past (*n* = 432)	395 (91)
WHO-5 ≤ 50 (*n* = 729)	510 (70)
**Work specific characteristics**	
Work Full Time (≥ 35 h/week vs. < 35 h/week)	495 (66)
**Company size (employees)**	
1–9	87 (11)
10–49	157 (21)
50–249	161 (21)
250–999	134 (18)
1000	216 (29)
**Occupational areas**	
Production of raw materials and goods and manufacturing	23 (3)
Construction, architecture, surveying and technical building services	20 (3)
Natural sciences, geography and informatics	59 (8)
Traffic, logistics, safety and security	27 (3)
Commercial services, trading, sales, the hotel business and tourism	111 (15)
Business organisation, accounting, law and administration	121 (16)
Health care, the social sector, teaching and education	292 (39)
Philology, literature, humanities, social sciences, economics, media, art, culture and design	83 (11)
Other	19 (2)
**Requirement levels**	
Unskilled or semi-skilled activities	95 (13)
Specialist activities	246 (33)
Complex specialist activities	239 (32)
Highly complex activities	175 (23)
Employed (vs. self-employed)	731 (97)
Without supervisor function (vs. with supervisor function)	603 (80)
Remote work	255 (34)
Shift work	145 (19)

^a^Categorised by ISCED 2011 – International Standard Classification of Education; *n* number, *SD* standard deviation; in case of missing data, the number of participants who answered the question are given

Regarding occupational areas, no participant belonged to the “Military” occupational area. Only few participants (*n* = 4) belonged to the “Agriculture, forestry, farming and horticulture” area. Therefore, these participants were assigned to the "Other" category.

### Preferences in general

Results of one-way RM ANOVAs to describe differences in agreement to implementation options regarding organisational aspects of psychotherapeutic consultation at work for the total study sample are presented in Table 3. Results of all post-hoc analyses can be found in Additional file 3. The results of all ANCOVA analyses can be viewed in Additional file 4. The analyses revealed significant within-subject differences in the agreement to implementation options of all organisational aspects. In more detail, employees preferred consultation outside company premises, a diagnostic session with combined treatment sessions and an accepted travel distance of up to 30 min. Consultation outside working hours was slightly preferred over consultation within working hours. Post-hoc analyses revealed that in-person consultation was preferred over video-based and telephone-based consultation. Video-based consultation was preferred over telephone-based consultation (t (df = 754) = -5,94, *p* < 0.001). Post-hoc analyses further showed that agreement to private burden as a purpose for consultation was smaller than agreement to occupational burden (t (df = 754) = 8.8, *p* < 0.001), to maintain the ability to work (t (df = 754) = 9.68, *p* < 0.001) or to occupational reintegration (t (df = 754) = -6.7, *p* < 0.001). No significant differences between the latter three consultation purposes were observed.

**Table 3:** Results of repeated measures analyses of variance (RM-ANOVA) for comparison between different implementation options of psychotherapeutic consultation at work (*n* = 755)

		**Effect of implementation options (within variable) on preference choices (repeated measures ANOVA)**	
	**mean (SD)**	**F (dfn, dfd)**	***p*-value**
**Type of consultation**			
In-person	4.78 (0.621)	F (2, 1508) = 752.054	** < 0.001**
Telephone	3.04 (1.21)		
Video-based	3.31 (1.24)		
**Location**			
Outside company premises	4.46 (0.851)	F (1,754) = 793.435	** < 0.001**
On company premises	2.60 (1.30)		
**Time**			
Outside working hours	3.62 (1.19)	F (1,754) = 5.413	**0.02**
Within working hours	3.44 (1.33)		
**Scope**			
Diagnostic only	2.84 (1.13)	F (1, 754) = 1012.71	** < 0.001**
Diagnostic + treatment	4.56 (0.743)		
**Purpose**			
Occupational burden	4.46 (0.922)	F (3, 2262) = 43.824	** < 0.001**
Maintain work ability	4.47 (0.885)		
Private burden	4.04 (1.05)		
Occupational reintegration	4.38 (1.03)		
**Accepted distance**	n (%)		
< 15 Min	99 (13)	n.a	
15 – 30 Min	466 (62)		
30 – 45 Min	149 (20)		
> 45 Min	28 (4)		
No transportation available	13 (2)		

*ANOVA* Analysis of variances, *n* number, *SD* standard deviation, *dfd* numerator degrees of freedom in the denominator, *dfn* degrees of freedom in the numerator

After adding covariates to the analyses, within-subject effects for all implementation options remained significant (*p* = 0.001—< 0.001). Significant effects of interactions between implementation options and covariates were found for all variables except for health insurance. Therefore age, gender, education, WHO-5, remote work and shift work were included in further analyses.

Subgroup analyses showed that employees with depressive symptoms preferred psychotherapeutic consultation to maintain work ability over psychotherapeutic consultation for occupational reintegration (t (df = 509) = 3.39, *p* = 0.005). All other results were similar to those of the total study sample (Additional file 5).

### Preferences for occupational areas, company size, supervisor function and requirement levels

Results of two-way RM ANOVAs to describe differences in agreement to implementation options between occupational areas, company size, supervisor function and requirement levels are shown in Tables 4, 5, 6. Those analyses revealed significant within-subject effects that are similar to those being described above. Generally all occupational groups, company sizes, requirement levels and employees with and without supervisor function preferred in-person consultation, consultation outside company premises and a diagnostic session being combined with further treatment sessions and accepted a travel distance of up to 30 min. Also results regarding location and purpose were similar for all those work-related characteristics. Consultation outside working hours was slightly preferred over consultation inside working hours. Furthermore, consultation for private burden was the least preferred purpose by participants of nearly all occupational areas, company sizes, requirement levels and by participants with and without supervisor function. Only few interaction effects between implementation options and work-related characteristics were observed. Post-hoc analyses revealed that those interaction effects were mainly describing differences in the level of agreement to specific implementation options, without changing the general preferences as mentioned above. Those interactions and post-hoc analyses are further described below.

**Table 4:** Results of analyses of variance for comparison between different implementation options depending on occupational area (*n* = 755)

	Production of raw materials and goods, and manu-facturing	Construction, architecture, surveying and technical building services	Natural sciences, geography and informatics	Traffic, logistics, safety and security	Commercial services, trading, sales, the hotel business and tourism	Business organisation, accounting, law and administration	Health care, the social sector, teaching and education	Philology, literature, humanities, social sciences, economics, media, art, culture, and design	Other	Effect of implementation options (within variable) and occupational areas (between variable) on preference choices (repeated measures ANOVA)		
										**F (dfn,dfd); (*p*-value)**		
	**mean (SD)**	**mean (SD)**	**mean (SD)**	**mean (SD)**	**mean (SD)**	**mean (SD)**	**mean (SD)**	**mean (SD)**	**mean (SD)**	**between**	**within**	**int**
**Type of consultation**										F (8, 746) =	F (2,1492) =	F (16, 1492) =
In-person	4.783 (0.85)	4.75 (0.64)	4.73 (0.72)	4.93 (0.27)	4.76 (0.65)	4.71 (0.64)	4.81 (0.59)	4.81 (0.61)	4.68 (0.58)	0.894; (0.521)	361.114; **(< 0.001)**	1.710; **(0.04)**
Telephone	3.043 (1.26)	2.75 (1.21)	3.08 (1.32)	3.11 (1.12)	2.77 (1.16)	3.13 (1.26)	3.12 (1.20)	3.05 (1.14)	2.89 (1.15)			
Video-based	3.391 (1.34)	3.10 (1.17)	3.58 (1.16)	2.85 (1.41)	3.25 (1.15)	3.35 (1.24)	3.24 (1.28)	3.59 (1.17)	3.42 (1.12)			
**Location**										F (8, 746) =	F (1, 746) =	F (8, 746) =
Outside company premises	4.35 (1.07)	4.50 (0.83)	4.56 (0.73)	4.67 (0.78)	4.59 (0.65)	4.36 (0.93)	4.35 (0.95)	4.61 (0.64)	4.84 (0.37)	0.557; (0.813)	439.310; (**< 0.001)**	2.563; (**0.009)**
On company premises	2.87 (1.42)	2.20 (1.24)	2.54 (1.25)	2.41 (1.22)	2.50 (1.29)	2.83 (1.38)	2.67 (1.30)	2.35 (1.14)	2.26 (1.41)			
**Time**										F (8, 746) =	F (1, 746) =	F (8, 746) =
Outside working hours	3.87 (1.36)	3.90 (1.12)	3.69 (1.09)	3.74 (1.32)	3.82 (1.23)	3.56 (1.12)	3.54 (1.21)	3.57 (1.16)	3.47 (1.22)	1.218; (0.285)	5.288; **(0.022)**	1.365; (0.208)
Within working hours	2.74 (1.48)	3.55 (1.36)	3.59 (1.34)	3.63 (1.28)	3.20 (1.37)	3.56 (1.31)	3.46 (1.33)	3.48 (1.22)	3.53 (1.17)			
**Scope**										F (8, 746) =	F (1, 746) =	F (8, 746) =
Diagnostic only	2.96 (1.40)	3.25 (1.12)	3.10 (1.16)	2.81 (1.24)	2.60 (0.99)	2.88 (1.14)	2.82 (1.15)	2.87 (1.13)	2.79 (0.92)	1.022; (0.418)	451.912; **(< 0.001)**	1.316; (0.232)
Diagnostic + treatment	4.52 (0.95)	4.45 (0.69)	4.56 (0.68)	4.78 (0.51)	4.61 (0.66)	4.52 (0.75)	4.54 (0.80)	4.54 (0.74)	4.79 (0.42)			
**Purpose**										F (8, 746) =	F (3, 2238) =	F (24, 2238) =
Occupational burden	4.17 (1.37)	4.65 (0.75)	4.58 (0.72)	4.26 (1.02)	4.44 (0.92)	4.40 (0.95)	4.53 (0.89)	4.40 (0.92)	4.05 (1.03)	0.631; (0.752)	15.802; **(< 0.001)**	1.045; (0.403)
Maintain work ability	4.35 (1.23)	4.55 (0.83)	4.34 (1.06)	4.59 (0.57)	4.46 (0.93)	4.51 (0.82)	4.49 (0.86)	4.36 (0.93)	4.58 (0.61)			
Private burden	3.91 (1.38)	4.10 (1.12)	4.03 (0.95)	4.11 (0.89)	4.11 (1.00)	3.93 (1.07)	4.02 (1.09)	4.13 (1.02)	4.16 (1.01)			
Occupational reintegration	4.26 (1.25)	4.60 (0.68)	4.34 (0.99)	4.33 (1.00)	4.27 (1.18)	4.36 (1.05)	4.46 (0.98)	4.41 (0.96)	3.89 (1.20)			
**Accepted distance**	n (%)	n (%)	n (%)	n (%)	n (%)	n (%)	n (%)	n (%)	n (%)	F (8, 733) =		
< 15 Min	4 (17)	1 (5)	9 (15)	4 (15)	12 (11)	18 (15)	39 (13)	10 (12)	2 (11)	0.48; (0.871)		
15 – 30 Min	10 (43)	15 (75)	32 (54)	18 (67)	73 (66)	82 (68)	171 (59)	51 (61)	14 (74)			
30 – 45 Min	5 (22)	4 (20)	15 (25)	4 (15)	18 (16)	18 (15)	66 (23)	17 (20)	2 (11)			
> 45 Min	1 (4)	0 (0)	3 (5)	1 (4)	4 (4)	3 (2)	11 (4)	4 (5)	1 (5)			

*ANOVA* Analysis of variances, *n* number, *SD* standard deviation, *dfd* numerator degrees of freedom in the denominator, *dfn* degrees of freedom in the numerator

**Table 5:** Results of analyses of variance for comparison between different implementation options depending on company size as well as supervisor function (*n* = 755)

	**Company size**								**Supervisor function**				
	1 – 9 employees	10 – 49 employees	50 – 249 employees	250 – 999 employees	≥ 1000 employees	Effect of implementation options (within variable) and company size (between variable) on preference choices (repeated measures ANOVA)			Without supervisor function	With supervisor function	Effect of implementation options (within variable) and supervisor function (between variable) on preference choices (repeated measures ANOVA)		
						**F (dfn,dfd); (*p*-value)**					**F (dfn,dfd); (*p*-value)**		
	**mean (SD)**	**mean (SD)**	**mean (SD)**	**mean (SD)**	**mean (SD)**	**Between**	**Within**	**Int**	**mean (SD)**	**mean (SD)**	**Between**	**Within**	**Int**
**Type of consultation**						F (4, 750) =	F (2, 1500) =	F (8, 1500) =			F (1, 753) =	F (2, 1506) =	F (2, 1506) =
In-person	4.76 (0.65)	4.75 (0.78)	4.79 (0.61)	4.72 (0.66)	4.83 (0.43)	0.394; (0.813)	686.865; (**< 0.001)**	0.881; (0.53)	4.80 (0.59)	4.70 (0.73)	0.218; (0.641)	464.805; **(< 0.001)**	0,510; (0.600)
Telephone	3.01 (1.23)	2.97 (1.16)	3.06 (1.24)	3.18 (1.10)	3.01 (1.27)				3.04 (1.22)	3.05 (1.13)			
Video-based	3.14 (1.26)	3.35 (1.20)	3.29 (1.25)	3.31 (1.20)	3.38 (1.27)				3.32 (1.24)	3.31 (1.21)			
**Location**						F (4, 750) =	F (1, 750) =	F (4, 750) =			F (1, 753) =	F (1, 753) =	F (1, 753) =
Outside company premises	4.48 (0.85)	4.52 (0.78)	4.42 (0.91)	4.41 (0.89)	4.46 (0.83)	3.456; **(0.008)**	760.786; **(< 0.001)**	2.828; **(0.024)**	4.45 (0.87)	4.49 (0.79)	5.092; (**0.024)**	570.146; **(< 0.001)**	4.297; **(0.039)**
On company premises	2.39 (1.22)	2.31 (1.21)	2.65 (1.29)	2.66 (1.32)	2.81 (1.35)				2.66 (1.29)	2.36 (1.29)			
**Time**						F (4, 750) =	F (1, 750) =	F ( 4, 750) =			F (1, 753) =	F (1, 753) =	F (1, 753) =
Outside working hours	3.66 (1.27)	3.50 (1.23)	3.61 (1.16)	3.66 (1.19)	3.68 (1.16)	1.411; (0.229)	5.338; (**0.021)**	0.826; (0.509)	3.60 (1.19)	3.73 (1.20)	0.218; (0.641)	8.008; **(0.005)**	2.593; (0.108)
Within working hours	3.29 (1.42)	3.55 (1.29)	3.32 (1.32)	3.58 (1.24)	3.42 (1.37)				3.48 (1.31)	3.29 (1.40)			
**Scope**						F (4, 750) =	F (1, 750) =	F (4, 750) =			F (1, 753) =	F (1, 753) =	F (1, 753) =
Diagnostic only	2.79 (1.14)	2.80 (1.09)	2.76 (1.18)	2.83 (1.11)	2.95 (1.15)	2.0640; (0.084)	928.921; (**< 0.001)**	0.325; (0.862)	2.81 (1.14)	2.94 (1.11)	0.073; (0.787)	601.966; **(< 0.001)**	2.909; (0.089)
Diagnostic + treatment	4.53 (0.70)	4.45 (0.89)	4.58 (0.78)	4.58 (0.70)	4.63 (0.63)				4.58 (0.72)	4.48 (0.84)			
**Purpose**						F (4, 750) =	F (3, 2250) =	F (12, 2250) =			F (1, 753) =	F (3, 2259) =	F (3, 2259) =
Occupational burden	4.28 (1.16)	4.31 (1.03)	4.50 (0.90)	4.53 (0.80)	4.56 (0.79)	1.170; (0.323)	32.755; (**< 0.001)**	2.684; (**0.002)**	4.47 (0.91)	4.40 (0.98)	2.894; (0.089)	32.552; **(< 0.001)**	0.497; (0.661)
Maintain work ability	4.32 (0.99)	4.41 (0.97)	4.48 (0.92)	4.51 (0.74)	4.53 (0.82)				4.48 (0.86)	4.43 (0.98)			
Private burden	4.15 (1.02)	3.99 (1.15)	4.18 (0.96)	4.10 (1.04)	3.88 (1.06)				4.07 (1.03)	3.90 (1.15)			
Occupational reintegration	4.30 (1.10)	4.34 (1.10)	4.30 (1.15)	4.34 (1.03)	4.53 (0.84)				4.40 (0.99)	4.29 (1.17)			
**Accepted distance**	**n (%)**	**n (%)**	**n (%)**	**n (%)**	**n (%)**	F (4, 737) =			**n (%)**	**n (%)**	F (1, 740) =		
< 15 Min	15 (17)	13 (8)	26 (17)	16 (12)	29 (14)	0.468; (0.759)			85 (14)	14 (9)	0.229; (0.633)		
15 – 30 Min	50 (57)	114 (75)	87 (56)	89 (67)	126 (59)				363 (61)	103 (69)			
30 – 45 Min	20 (23)	21 (14)	35 (22)	24 (18)	49 (23)				122 (21)	27 (18)			
> 45 Min	2 (2)	5 (3)	8 (5)	3 (2)	10 (5)				22 (4)	6 (4)			

*ANOVA* Analysis of variances, *n* number, *SD* standard deviation, *dfd* numerator degrees of freedom in the denominator, *dfn* degrees of freedom in the numerator

**Table 6:** Results of analyses of variance for comparison between different implementation options depending on requirement level (*n *= 755)

	Unskilled or semiskilled activities	Specialist activities	Complex specialist activities	Highly complex activities	Effect of implementation options (within variable) and requirement levels (between variable) on preference choices (repeated measures ANOVA)		
					**F (dfn,dfd); (*p*-value)**		
	**mean (SD)**	**mean (SD)**	**mean (SD)**	**mean (SD)**	**Between**	**Within**	**Int**
**Type of consultation**					F (3, 751) =	F (2, 1502) =	F (6, 1502) =
In person	4.88 (0.35)	4.78 (0.63)	4.74 (0.67)	4.75 (0.65)	2.7722; (**0.041)**	685.960; **(< 0.001)**	3.725; **(0.001)**
Telephone	2.80 (1.27)	3.02 (1.17)	3.08 (1.19)	3.15 (1.24)			
Video-based	3.15 (1.28)	3.16 (1.21)	3.32 (1.26)	3.61 (1.17)			
**Location**					F (3, 751) =	F (1, 751) =	F (3, 751) =
Outside company premises	4.57 (0.72)	4.43 (0.90)	4.43 (0.86)	4.47 (0.84)	1.308; (0.27)	690.093; **(< 0.001)**	0.284; (0.84)
On company premises	2.71 (1.32)	2.64 (1.33)	2.57 (1.29)	2.52 (1.27)			
**Time**					F (3, 751) =	F (1, 751) =	F (3, 751) =
Outside working hours	3.52 (1.33)	3.58 (1.17)	3.69 (1.14)	3.65 (1.23)	0.551; (0.647)	4.085; **(0.044)**	0.390; (0.760)
Within working hours	3.41 (1.31)	3.47 (1.30)	3.39 (1.36)	3.47 (1.34)			
**Scope**					F (3, 751) =	F ( 1, 751) =	F (3, 751) =
Diagnostic only	2.74 (1.05)	2.80 (1.12)	2.80 (1.11)	2.99 (1.23)	1.000; 0.392	881.059; **(< 0.001)**	1.128; (0.337)
Diagnostic + treatment	4.57 (0.68)	4.59 (0.64)	4.54 (0.81)	4.54 (0.81)			
**Purpose**					F (3, 751) =	F ( 3, 2253) =	F (9, 2253) =
Occupational burden	4.19 (1.12)	4.45 (0.93)	4.56 (0.82)	4.47 (0.90)	1.934; (0.123)	30.878; **(< 0.001)**	1.915; (0.054)
Maintain work ability	4.38 (0.91)	4.52 (0.82)	4.53 (0.80)	4.35 (1.05)			
Private burden	4.17 (1.02)	4.05 (1.03)	4.04 (1.02)	3.94 (1.15)			
Occupational reintegration	4.26 (1,07)	4.35 (1,07)	4.49 (0,99)	4.34 (1,01)			
**Accepted distance**	n (%)	n (%)	n (%)	n (%)	F (3, 738) =		
< 15 Min	13 (14)	39 (16)	24 (10)	23 (13)	0.214; (0.887)		
15 – 30 Min	58 (64)	144 (59)	156 (67)	108 (62)			
30 – 45 Min	16 (18)	50 (21)	42 (18)	41 (23)			
> 45 Min	4 (4)	10 (4)	11 (5)	3 (2)			

*ANOVA* Analysis of variances, *n* number, *SD* standard deviation, *dfd* numerator degrees of freedom in the denominator, *dfn* degrees of freedom in the numerator

### Occupational areas

There was a significant interaction between occupational area and type of consultation and location of consultation (Table 4). Post-hoc analyses revealed a significant preference of video-based consultation over telephone-based consultation in the occupational areas of „Natural sciences, geography and informatics” (t (df = 58) = -3.1; *p* = 0.008), „Commercial services, trading, sales, the hotel business and tourism” (t (df = 110) = -4.5; *p* < 0.001) and „ Philology, literature, humanities, social sciences, economics, media, art, culture, and design” (t (df = 82) = -4.0; *p* < 0.001). For other occupational areas, no difference in preference for video-based and telephone-based consultation existed. In all occupational areas, implementation outside premises was significantly preferred over implementation on company premises. 

After controlling for covariates (age, gender, WHO-5, remote work, education and shift work), the interaction between occupational area and type of consultation (F (16, 1426) = 1.108, *p* = 0.342) as well as the within-subject effect of time of consultation was no longer significant (F (1, 713) = 0.238, *p* = 0.626). No other differences to the uncontrolled analyses were observed.

### Company size

There was a significant interaction between company size and location of consultation (Table 5). Post-hoc analyses revealed that employees of large companies (≥ 1000 employees) indicated significantly higher acceptance scores regarding implementation on company premises compared to smaller companies (10–49 employees; t (df = 355) = -3.8; *p* = 0.002). There was a significant difference between company sizes in terms of why employees would seek psychotherapeutic consultation at the work. Seeking consultation at work for private burden was significantly less preferred compared to the other reasons by employees of all company sizes (*p* = 0.035—< 0.001, see Additional file 3). Only participants from companies with 1–9 employees showed no significant differences between all purposes (*p* = 1). Furthermore, participants from companies with 50–249 employees showed no significant difference between private burden and reintegration (t (df = 160) = -1.0; *p* = 1). Employees from large companies (> 1000 employees) would be less likely to seek consultation for private burden than employees from middle-sized companies with 50–249 employees (t (df = 361) = 2.8; *p* = 0.05).

Similar results were obtained for the ANCOVA analyses after controlling for covariates.

### Supervisor function

There was a significant interaction between supervisor function and location of consultation (Table 5). Post-hoc analyses showed that employees with supervisor function disapproved consultation on company premises significantly more than employees without supervisor function (t (df = 233) = 2.5; *p* = 0.012, see Additional file 3).

The interaction between supervisor function and location of consultation remained significant after controlling for covariates (F (1, 720) = 4.025, *p* = 0.045).

### Requirement levels

There was only a significant interaction between requirement levels and type of consultation (Table 6). Post-hoc analyses revealed that employees performing highly complex activities indicated significant higher acceptance of video-based consultation compared to employees performing unskilled or semiskilled activities (t (df = 179) = -2.9); *p* = 0.026) and specialist activities (t (df = 383) = -3.8). Video-based implementation was preferred over telephone-based implementation within all requirement levels except in the specialised activities group.

After controlling for covariates, the interaction between requirement levels and type of consultation was no longer significant (F (6, 1436) = 1.159, *p* = 0.326).

### Discussion

This study showed that employees prefer in-person consultation, which takes place outside company premises and outside working hours and includes a diagnostic session with further treatment sessions. In general, participants agreed to all occupational purposes, but agreement to occupational aspects was stronger than for private aspects. Most participants indicated that the one-way distance to the consultation should not exceed 15 to 30 min. Within some implementation options, the magnitude of agreement differed between occupational area, company size, supervisor function and level of requirement, but this did not affect the abovementioned key results.

Within two previous qualitative studies, company stakeholders (e.g. occupational physicians), psychotherapists and employees who had used psychotherapeutic consultation at work expressed that conducting consultation outside company premises and outside working hours could increase anonymity, counteract fear of stigmatisation and promote more flexible appointment scheduling [20]. One might consequently assume that higher anonymity is one of the reasons why in our study especially employees of smaller companies and employees with supervisor function prefer consultation outside company premises over consultation on company premises. Employees with supervisor function may have concerns about being seen by their subordinates and might therefore especially value consultation outside company premises. In larger companies, there might be a greater chance that colleagues would not notice when someone is attending psychotherapeutic consultations on company premises. Additionally, larger companies often have their own facilities for occupational health issues. If those facilities also host psychotherapeutic consultation at work, colleagues might consequently not be able to deduce the reason on why someone is visiting these facilities. However, the above-mentioned qualitative studies further stated that conducting consultation on company premises and within working hours could be low-threshold, as sessions could be easily combined with employees ‘ working hours [20]. It was further presumed by the participants in that study that the distance to the consultation location could have a negative impact on utilisation [20]. However, if consultation is conducted outside company premises, the majority of participants in our study indicated that they would accept a distance of up to 30 min travel time. However, feasibility of this aspect needs further exploration, because especially rural regions are characterised by a lack of psychotherapists [36, 59]. A previous study analysing practical experience of psychotherapeutic consultation at work in two big German companies found that employees also make use of a psychotherapeutic consultation at work if it takes place on company premises [35].

In previous studies, digital solutions such as video-based consultation are recommended as an alternative for people living in rural regions or people who have no means of transport available [36, 59, 60]. However, within our study, participants rather preferred in-person than video-based consultation. Therefore, it should be further investigated whether video-based implementation of psychotherapeutic consultation at work would be accepted as an alternative when in-person implementation is not possible at a certain distance. Also other studies found a preference for in-person consultation over video-based and telephone-based consultation [29, 58]. Our study further adds that there is higher acceptance towards video-based consultation among employees with highly complex work tasks compared to those with lower requirement levels. This might be due to the fact that people with higher job requirement levels might be more likely to have office jobs and therefore be more accustomed to video-based communication. In addition, the COVID-19 pandemic has led to an increase in in-person meetings being converted to video meetings [61, 62]. As this study took place during the Covid-19 pandemic, this aspect might also have influenced the results. Nevertheless, these employees still preferred in-person consultation over video-based consultation.

On average, employees from our study indicated that they would rather use psychotherapeutic consultation at work for occupational than private burden. Our study results further suggests that employees from larger companies would be less likely to use psychotherapeutic consultation at work to talk about private burden than employees from smaller companies. The preference for discussing occupational burden rather than private burden was also found in a previous study questioning employees from one single German company [29]. In that study, a quarter of the participants indicated that they would not visit psychotherapeutic consultation at work because of private burden. However, separating and only discussing occupational or private problems during psychotherapeutic consultation at work might not be feasible, as discussed by previous studies [23, 35]. In one previous study, occupational burden was apparent in 80% of the participants who made use of psychotherapeutic consultation at work but in only 40% of cases workplace-related difficulties were classified as the main cause for the development of psychological burden [35]. In the present study, employees with mental distress slightly preferred psychotherapeutic consultation at work for the purpose of maintaining work ability over the purpose of reintegration. This difference could not be found in the total study sample. So far, there is no comparative literature that can provide a potential rationale for this. It can be discussed whether maintaining work ability was currently considered more relevant than reintegration by the participants with depressive symptoms in the present study and was therefore rated more positively since despite the high prevalence of depressive symptoms, only a minority was currently unable to work due to mental illness. However, these participants also agreed on average that they would seek psychotherapeutic consultation at work for reintegration.

On average, the option of offering a diagnostic session only was rejected, while offering additional treatment sessions afterwards was significantly preferred. Those study results support current practice offering ten or more treatment sessions after a diagnostic session [21, 35]. However, the number of appropriate sessions could not be determined within this study and clinical studies are necessary to determine which scope is effective at a clinical and economic level. To a certain extent some flexibility would be advisable to allow psychotherapists to adjust the number of sessions to the individual need of the employee [63–65]. In this context, the aim of psychotherapeutic consultation at work should also be considered and explained to the employee. On the one hand, models of psychotherapeutic consultation at work often include comprehensive clinical as well as work-related diagnostics with following treatment sessions if needed and thereby differ from coaching or counselling as offered by EAP [21, 25, 66]. Based on those comprehensive diagnostics, short-term psychotherapy for employees with mental illnesses or psychotherapeutic prevention for employees with subclinical symptoms could be provided [21]. Therefore, healthy employees with current work-related problems could benefit from psychotherapeutic consultation at work by offering primary prevention as well as employees who already developed mental illnesses by offering secondary prevention [21]. On the other hand, most models of psychotherapeutic consultation at work are offering only a limited number of sessions and are therefore not intended to replace standard psychotherapeutic care [21, 22, 35]. Psychotherapeutic consultation at work rather improves access by providing a short-term service and to bridge waiting time until therapy in standard care [21]. For this reason, the possibilities of psychotherapeutic consultation at work should be discussed with the employee in order to avoid possible side effects that may arise, for example, due to false expectations of the sessions [67, 68]. Stakeholders being involved in the implementation of psychotherapeutic consultation at work (e.g. managers, occupational physicians, psychotherapists) should be aware not to cause dysfunctional sensitization for mental health problems in employees who have "work load" but no "mental illness" [67]. Work demands and mental disorders are two different things and healthy suffering due to work load must not be misunderstood as mental disorder [67]. Therefore, prevention of mental illness should not be confused with psychotherapeutic treatment. A qualitative study with managers and employees raised concerns that sensitisation effects could arise when speaking too much about mental health in mentally healthy teams [67]. However, a longitudinal study showed that an awareness campaign in a company did not lead to increased levels of reported psychological distress but to a reduction in stigma and increased likelihood of help-seeking for mental health issues [58].

Previous studies have reported that occupational factors such as shift work, remote work or other specific aspects may have an impact on the implementation of mental health interventions [39, 58]. Our study therefore included shift work and remote work in the ANCOVA analyses. However, this had no effect on the main results in terms of type, time, location, scope, purpose and distance.

Furthermore, previous research indicates that small companies are less likely to offer health interventions [38, 43], but that small companies are motivated to implement health interventions when given a specific implementation guideline [38]. Our study suggests that preferences regarding organisational aspects of psychotherapeutic consultation at work are very similar between employees of smaller and larger companies. The results therefore demonstrate that an implementation guideline with the organisational aspects considered in this study might be applied regardless of company size.

### Strengths & limitations

One strength of this study is the broad composition of the study population in terms of occupational area, company size, requirement level and supervisor function. This allowed us to identify preferences of a wide variety of employees working in different employment settings.

Furthermore, a large number of participants stated that they had recently or in the past been diagnosed with a mental illness. This was also reflected by the WHO-5 with 70% of participants suffering from current symptoms of depression. The study therefore specifically reflects the perspectives of one relevant target group for psychotherapeutic consultation at work. However, psychotherapeutic consultation at work is also intended to reach employees with subclinical symptoms of mental illnesses. We have therefore tried to reach employees with and without mental illness by our advertisements. However, the slogan of our advertisement might have primarily addressed people who are currently suffering or have suffered in the past from mental illnesses. In order to nevertheless take into account preferences of participants without current depression, subgroup analyses for employees with current symptoms of depression were performed. In addition, we controlled for depressive symptoms in the ANCOVA analyses. However, results of subgroup and ANCOVA analyses suggest that opinions on organisational aspects of psychotherapeutic consultation are similar between employees with and without depressive symptoms.

A limitation of this study is that the study population was predominantly female and of younger age. Overrepresentation of younger and female participants due to recruitment via Facebook and Instagram had already been assumed in advance [50]. LinkedIn was therefore used as a third medium to recruit participants and achieve a more balanced gender proportion. However, due to the very low response rate on LinkedIn, this had no effect on the gender proportion of the overall study population.

Furthermore, one might consider that the study sample was composed by people who were active users of Facebook, Instagram and/or LinkedIn. Those might differ from people who are not registered in or use these social media platforms. For example, they might have a greater affinity for digital communication and are therefore more likely to also use video-based consultation.

The availability of psychotherapeutic services varies regionally (e.g. between urban and rural areas [36, 37]). This might have an influence on the preferences of some organisational aspects of psychotherapeutic consultation including type, location or accepted travel distance. However, this aspect was not considered in this study and should therefore be taken into account in future studies.

Furthermore, two other limitations need to be considered when interpreting the results of the accepted travel distance to psychotherapeutic consultation at work. First, from results of this study, it is not possible to determine which participants assumed travel distance from their workplace or from home. Therefore, it cannot be generally concluded that the consultation location should be located within 15–30 min from company premises, as this might lead to longer travel distances from the home address than accepted. Second, it is not clear whether participants assumed to travel by car or by public transport. However, such information needs to be taken into account with regard to regional infrastructure.

We cannot rule out the possibility that preference differences within specific occupations were missed within the analysis, because a rather broad categorisation of occupational areas was used. The German classification code of 2010 [41] offers opportunities for a more specific categorization of occupations. However, due to its complexity, more specific categorization is hardly feasible within a short online questionnaire.

### Implications

From the results of this study one might derive that psychotherapeutic consultation at work should be offered as in-person consultation including diagnostic as well as treatment sessions outside company premises but in a reasonable distance of up to 30 min travel time from employees’ work or home locations. The results further suggest that psychotherapeutic consultation at work will be more accepted if offered outside working hours and if consultation focuses on occupational aspects including reintegration, maintaining work ability and discussing occupational burden. However, specification of time and purpose seems to be less important for employees than type, scope and location of consultation. The results of this study further suggest that no specific adaptations of those organisational aspects might be needed for different occupational areas, company sizes, requirement levels or for employees with vs. without supervisor function.

Supervisors have an important role in the implementation of mental health interventions at work [47, 58]. By creating a supportive atmosphere towards openness and treatment of mental illness, utilisation of treatment among employees can be increased [47, 58]. Thus, supervisors can indirectly influence utilisation by creating this atmosphere and directly by recommending the treatment service to employees. Therefore supervisors’ approval is crucial to involve them in the implementation and promotion of psychotherapeutic consultation at work [58]. Our results thereby suggest that approval of supervisors can be achieved by the same implementation options which employees without supervisor function would prefer.

This study focused on psychotherapeutic consultation at work. However, the results of this study might also be transferred to other concepts that share similar organisational aspects and offer psychological support for employees experiencing mental distress (e.g. EAP).

Regarding future research, more organisational aspects than those discussed in our study need to be considered. These include, inter alia, processes to select responsible persons within the company and financing approaches (e.g. by the company or health insurance) [20, 23, 43, 69]. Further research is needed to determine whether preferences on these aspects differ between occupational areas, company sizes, supervisor function and requirement levels. Especially among small companies, financial barriers were mentioned regarding implementation of health interventions [43, 44] and should therefore be considered in further analyses. This study theoretically investigated how psychotherapeutic consultation at work should be implemented in order that employees make use of it. To analyse whether employees actually make use of it under these conditions, additional analyses are needed. Further, this study focussed on the preferences of employees as potential users of psychotherapeutic consultation at work. As the perspective of psychotherapists and companies involved has only been investigated in qualitative studies so far [20], quantitative studies might be required to examine preferences of these and potentially other stakeholders involved.

## Conclusion

The results of this study give practical indications which organisational implementation options should be considered regarding psychotherapeutic consultation at work. Accordingly, consultation should be provided in-person and outside company premises and should include further treatment sessions after a diagnostic session in order to be accepted by employees. Specification of time and purpose options seemed to be less important. The findings suggest that those options will be accepted by employees regardless of occupational area, company size, supervisor function and requirement level. Nevertheless, there are further aspects to be considered regarding implementation of psychotherapeutic consultation at work including personnel and financial issues. Taking employees’ preferences into account may potentially have a positive effect on the utilisation of psychotherapeutic consultation at work and thus on the psychotherapeutic care of individuals with mental disorders. However, this needs to be analysed during practical implementation by future research.

## Supplementary Information

**Additional file 1.** Recruitment process.

**Additional file 2.** Questionnaire on preferences.

**Additional file 3.** Results of post-hoc analyses.

**Additional file 4.** Results of ANCOVA analyses.

**Additional file 5.** Results of sensitivity analyses.

## Data Availability

The data sets used and/or analysed in this study can be handed out in anonymised form. Please contact the author Fiona Kohl (fiona.kohl@uni-duesseldorf.de) in this regard.
