# Supplementary material for: Employees’ preferences on organisational aspects of psychotherapeutic consultation at work by occupational area, company size, requirement levels and supervisor function – a cross-sectional study in Germany
Source: BMC Public Health. 2023 Feb 16;23:347. doi: 10.1186/s12889-023-15255-0 (PMC9932407; doi:10.1186/s12889-023-15255-0)

Employees' preferences on organisational aspects of psychotherapeutic consultation at work by occupational area, company size, requirement levels and supervisor function – a cross-sectional study in Germany

Fiona Kohl[1], Peter Angerer[1], Jeannette Weber[1]

[1] Institute of Occupational, Social and Environmental Medicine, Centre for Health and Society, Medical Faculty, Heinrich-Heine-University Düsseldorf, Moorenstraße 5, 40225 Düsseldorf, Germany
Corresponding author: Jeannette Weber, Institute of Occupational, Social and Environmental Medicine, Centre for Health and Society, Medical Faculty, Heinrich-Heine-University Düsseldorf, Moorenstraße 5, 40225 Düsseldorf, Germany, Email: Jeannette.Weber@hhu.de

Additional file 1 – Recruitment process

Figure 1 Description of the recruitment process in the study

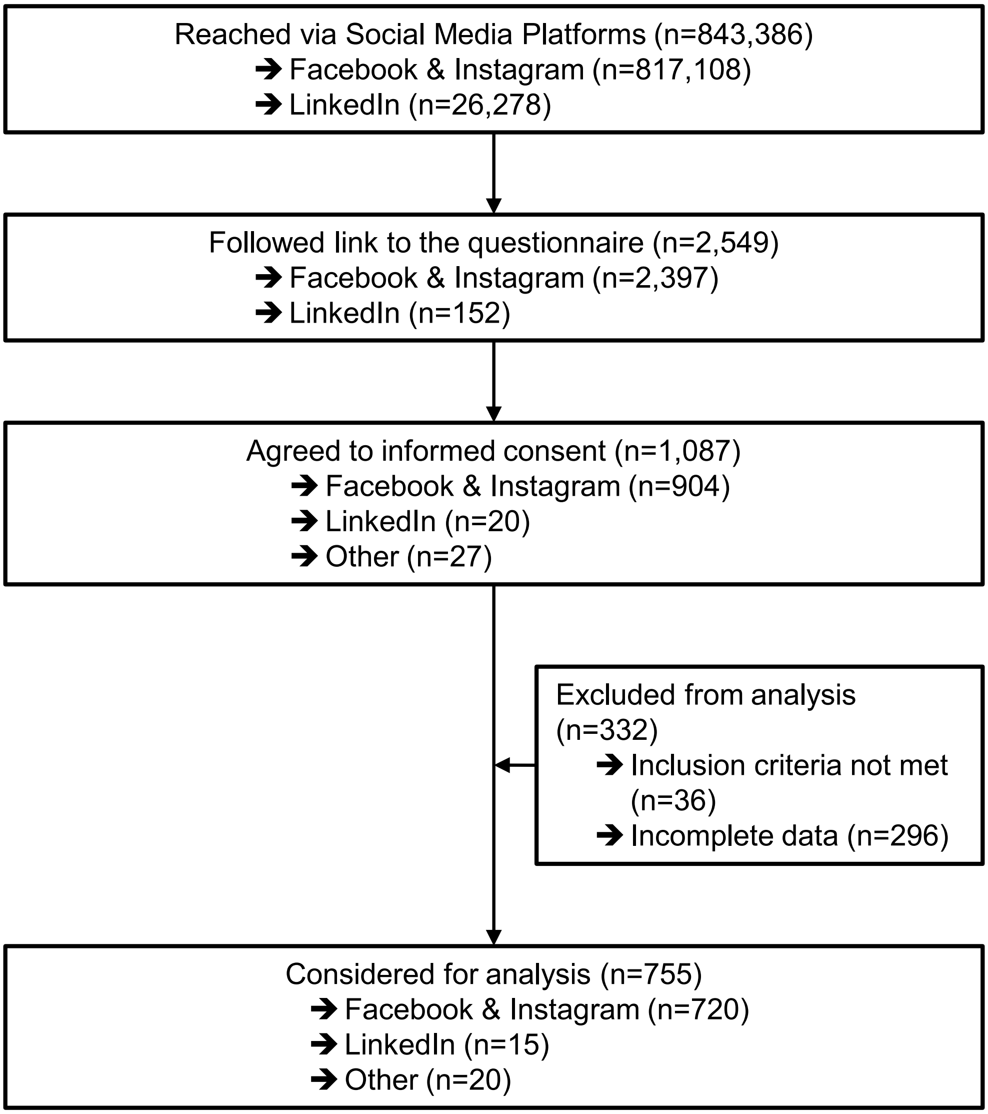

Supplement: Supplementary file 1 — Additional file 1. Recruitment process. [file 12889_2023_15255_MOESM1_ESM.pdf]
