# Supplementary material for: Employees’ preferences on organisational aspects of psychotherapeutic consultation at work by occupational area, company size, requirement levels and supervisor function – a cross-sectional study in Germany
Source: BMC Public Health. 2023 Feb 16;23:347. doi: 10.1186/s12889-023-15255-0 (PMC9932407; doi:10.1186/s12889-023-15255-0)
Supplement: Supplementary file 2 — Additional file 2. Questionnaire on preferences. [file 12889_2023_15255_MOESM2_ESM.docx]

**Employees’ preferences on organisational aspects of psychotherapeutic consultation at work by occupational area, company size, requirement levels and supervisor function – a cross-sectional study in Germany**

Fiona Kohl^1^, Peter Angerer^1^, Jeannette Weber^1^

^1^ Institute of Occupational, Social and Environmental Medicine, Centre for Health and Society, Medical Faculty, Heinrich-Heine-University Düsseldorf, Moorenstraße 5, 40225 Düsseldorf, Germany

Corresponding author: Jeannette Weber, Institute of Occupational, Social and Environmental Medicine, Centre for Health and Society, Medical Faculty, Heinrich-Heine-University Düsseldorf, Moorenstraße 5, 40225 Düsseldorf, Germany, Email: Jeannette.Weber@hhu.de

**Additional file 2 – Questions used regarding preferences for implementation options regarding psychotherapeutic consultation at work**

*Imagine that your employer gives you the opportunity to receive psychotherapeutic consultation. Professionally trained psychotherapists who are bound by medical confidentiality (also towards your employer) would conduct this consultation. For you as an employee, there would be no costs for the entire treatment. We are now very interested in your opinion on such consultation to draw conclusions on how it could best be implemented. Please answer the questions even if you are not currently affected by a mental illness.*

In the following, we ask you whether you would consider to use psychotherapeutic consultation at work and how it should be organised.

| Now imagine that psychotherapeutic consultation at work should be implemented in your company. How should it be structured?  *Please state your opinion for each answer option.* | | | | | | | | | |
| --- | --- | --- | --- | --- | --- | --- | --- | --- | --- |
|  | | Strongly disagree | Disagree | | Neither agree nor disagree | | | Agree | Strongly agree |
| 1. What type of individual consultation should be conducted? | | | | | | | | | |
| - 1. In-person | | 🞏 | | 🞏 | | 🞏 | 🞏 | | 🞏 |
| - 1. Telephone-based | | 🞏 | | 🞏 | | 🞏 | 🞏 | | 🞏 |
| - 1. Video-based | | 🞏 | | 🞏 | | 🞏 | 🞏 | | 🞏 |
| 1. At which location should the consultation be conducted? | | | | | | | | | |
| - 1. On premises of my company (e.g. the company medical service) | | 🞏 | | 🞏 | | 🞏 | 🞏 | | 🞏 |
| - 1. Outside company premises (e.g. in a psychotherapist’s practice) | | 🞏 | | 🞏 | | 🞏 | 🞏 | | 🞏 |
| 1. At what time should the consultation be conducted? | | | | | | | | | |
| - 1. Within working hours | | 🞏 | | 🞏 | | 🞏 | 🞏 | | 🞏 |
| - 1. Outside working hours | | 🞏 | | 🞏 | | 🞏 | 🞏 | | 🞏 |
| 1. Which scope should the consultation include? | | | | | | | | | |
| - 1. Only one diagnostic session for problem analysis and orientation | | 🞏 | | 🞏 | | 🞏 | 🞏 | | 🞏 |
| - 1. Diagnostic discussion with the possibility of further treatment (max. 10 sessions) | | 🞏 | | 🞏 | | 🞏 | 🞏 | | 🞏 |
| 1. For what purpose would you attend the consultation if you were/are affected by a mental illness? | | | | | | | | | |
| - 1. Occupational burden | | 🞏 | | 🞏 | | 🞏 | 🞏 | | 🞏 |
| - 1. Private burden | | 🞏 | | 🞏 | | 🞏 | 🞏 | | 🞏 |
| - 1. To maintain the ability to work in the presence/threat of mental illness | | 🞏 | | 🞏 | | 🞏 | 🞏 | | 🞏 |
| - 1. On reintegration after absence due to mental illness | | 🞏 | | 🞏 | | 🞏 | 🞏 | | 🞏 |
| 1. What do you consider to be the maximum acceptable one-way distance to get to the place of the consultation? (Start from work or home) | | | | | | | | | |
| 🞏 | < 15 min | | | | | | | | |
| 🞏 | 15 – 30 min. | | | | | | | | |
| 🞏 | 30 – 45 min. | | | | | | | | |
| 🞏 | > 45 min. | | | | | | | | |
| 🞏 | I do not have transport means available (e.g. car, train), so I would not be able to travel to consultation. | | | | | | | | |
