# Supplementary material for: Employees’ preferences on organisational aspects of psychotherapeutic consultation at work by occupational area, company size, requirement levels and supervisor function – a cross-sectional study in Germany
Source: BMC Public Health. 2023 Feb 16;23:347. doi: 10.1186/s12889-023-15255-0 (PMC9932407; doi:10.1186/s12889-023-15255-0)
Supplement: Supplementary file 3 — Additional file 3. Results of post-hoc analyses. [file 12889_2023_15255_MOESM3_ESM.docx]

**Employees’ preferences on organisational aspects of psychotherapeutic consultation at work by occupational area, company size, requirement levels and supervisor function – a cross-sectional study in Germany**

Fiona Kohl^1^, Peter Angerer^1^, Jeannette Weber^1^

^1^ Institute of Occupational, Social and Environmental Medicine, Centre for Health and Society, Medical Faculty, Heinrich-Heine-University Düsseldorf, Moorenstraße 5, 40225 Düsseldorf, Germany

Corresponding author: Jeannette Weber, Institute of Occupational, Social and Environmental Medicine, Centre for Health and Society, Medical Faculty, Heinrich-Heine-University Düsseldorf, Moorenstraße 5, 40225 Düsseldorf, Germany, Email: Jeannette.Weber@hhu.de

**Additional file 3 – Results of post-hoc analyses**

**General sample**

Table 1 Results of t-tests for dependent samples with Bonferroni correction method to analyse differences of agreement to different implementation options regarding type of consultation at work (n=755)

| **Purpose of consultation** | **Purpose of consultation** | **n1** | **n2** | **Statistic (t)** | **df** | **p** | **p.adj** | **p.adj.signif** |
| --- | --- | --- | --- | --- | --- | --- | --- | --- |
| type_person | type_telephone | 755 | 755 | 35.1 | 754 | <0.001 | <0.001 | **** |
| type_person | type_video | 755 | 755 | 29.80 | 754 | <0.001 | <0.001 | **** |
| type_telephone | type_video | 755 | 755 | -5.94 | 754 | <0.001 | <0.001 | **** |

| *df = degree of freedom; n = number; *p ≤ .05; **p < .01; ***p < .001; ns = not significant (p > .05)* |
| --- |

Table 2 Results of t-tests for dependent samples with Bonferroni correction method to analyse differences of agreement to different implementation options regarding purpose of consultation at work (n=755)

| **Purpose of consultation** | **I Purpose of consultation** | **n1** | **n2** | **Statistic (t)** | **df** | **p** | **p.adj** | **p.adj.signif** |
| --- | --- | --- | --- | --- | --- | --- | --- | --- |
| purpose_occupational | content_maintain | 755 | 755 | -0.252 | 754 | 0.801 | 1 | ns |
| purpose_occupational | content_private | 755 | 755 | 8.8 | 754 | <0.001 | <0.001 | **** |
| purpose_occupational | content_reintegration | 755 | 755 | 1.86 | 754 | 0.063 | 0.379 | ns |
| purpose_maintain | content_private | 755 | 755 | 9.68 | 754 | <0.001 | <0.001 | **** |
| purpose_maintain | content_reintegration | 755 | 755 | 2.4 | 754 | 0.017 | 0.101 | ns |
| purpose_privat | content_reintegration | 755 | 755 | -6.7 | 754 | <0.001 | <0.001 | **** |

| *df = degree of freedom; n = number; *p ≤ .05; **p < .01; ***p < .001; ns = not significant (p > .05)* |
| --- |

**Occupational area**

Table 3 Results of pairwise t-tests for independent samples with Bonferroni correction method to analyse differences of agreement to different implementation options of type of consultation at work between different occupational areas (n=755)

| **Type of consultation** | **Occupational area** | **Occupational area** | **n1** | **n2** | **Statistic (t)** | **df** | **p** | **p.adj** | **p.adj.signif** |
| --- | --- | --- | --- | --- | --- | --- | --- | --- | --- |
| type_person | Production | Construction | 23 | 20 | 0.14321435 | 40.2071602 | 0.887 | 1 | ns |
| type_person | Production | Natural science | 23 | 59 | 0.26858502 | 34.7956784 | 0.79 | 1 | ns |
| type_person | Production | Traffic | 23 | 27 | -0.77625807 | 25.6926178 | 0.445 | 1 | ns |
| type_person | Production | Services | 23 | 111 | 0.13769056 | 27.5616224 | 0.891 | 1 | ns |
| type_person | Production | Business organisation | 23 | 121 | 0.38515426 | 26.9050818 | 0.703 | 1 | ns |
| type_person | Production | Health & social services | 23 | 292 | -0.14175555 | 23.6963151 | 0.888 | 1 | ns |
| type_person | Production | Humanities & social science | 23 | 83 | -0.12977597 | 28.6539001 | 0.898 | 1 | ns |
| type_person | Production | Other | 23 | 19 | 0.4431615 | 38.789084 | 0.66 | 1 | ns |
| type_person | Construction | Natural science | 20 | 59 | 0.12427726 | 36.4264682 | 0.902 | 1 | ns |
| type_person | Construction | Traffic | 20 | 27 | -1.1591974 | 23.9403073 | 0.258 | 1 | ns |
| type_person | Construction | Services | 20 | 111 | -0.04343627 | 26.5864376 | 0.966 | 1 | ns |
| type_person | Construction | Business organisation | 20 | 121 | 0.25466966 | 25.6786602 | 0.801 | 1 | ns |
| type_person | Construction | Health & social services | 20 | 292 | -0.39625585 | 21.2805135 | 0.696 | 1 | ns |
| type_person | Construction | Humanities & social science | 20 | 83 | -0.36240327 | 28.0832679 | 0.72 | 1 | ns |
| type_person | Construction | Other | 20 | 19 | 0.33640406 | 36.9424198 | 0.738 | 1 | ns |
| type_person | Natural science | Traffic | 59 | 27 | -1.85380896 | 81.7785757 | 0.067 | 1 | ns |
| type_person | Natural science | Services | 59 | 111 | -0.25022636 | 108.994966 | 0.803 | 1 | ns |
| type_person | Natural science | Business organisation | 59 | 121 | 0.16472133 | 104.200306 | 0.869 | 1 | ns |
| type_person | Natural science | Health & social services | 59 | 292 | -0.79969663 | 74.7629958 | 0.426 | 1 | ns |
| type_person | Natural science | Humanities & social science | 59 | 83 | -0.68225566 | 112.806534 | 0.496 | 1 | ns |
| type_person | Natural science | Other | 59 | 19 | 0.27389407 | 37.0149122 | 0.786 | 1 | ns |
| type_person | Traffic | Services | 27 | 111 | 2.10790869 | 103.938953 | 0.037 | 1 | ns |
| type_person | Traffic | Business organisation | 27 | 121 | 2.77704442 | 99.5632684 | 0.007 | 0.236 | ns |
| type_person | Traffic | Health & social services | 27 | 292 | 1.90204815 | 53.8154719 | 0.062 | 1 | ns |
| type_person | Traffic | Humanities & social science | 27 | 83 | 1.40076767 | 99.2877723 | 0.164 | 1 | ns |
| type_person | Traffic | Other | 27 | 19 | 1.68864147 | 23.359691 | 0.105 | 1 | ns |
| type_person | Services | Business organisation | 111 | 121 | 0.54344139 | 227.515806 | 0.587 | 1 | ns |
| type_person | Services | Health & social services | 111 | 292 | -0.72818843 | 182.952207 | 0.467 | 1 | ns |
| type_person | Services | Humanities & social science | 111 | 83 | -0.55250047 | 181.789776 | 0.581 | 1 | ns |
| type_person | Services | Other | 111 | 19 | 0.4929934 | 26.2904439 | 0.626 | 1 | ns |
| type_person | Business organisation | Health & social services | 121 | 292 | -1.44383283 | 209.194057 | 0.15 | 1 | ns |
| type_person | Business organisation | Humanities & social science | 121 | 83 | -1.0849567 | 180.738773 | 0.279 | 1 | ns |
| type_person | Business organisation | Other | 121 | 19 | 0.18215498 | 25.2932794 | 0.857 | 1 | ns |
| type_person | Health & social services | Humanities & social science | 292 | 83 | 0.01307728 | 128.18269 | 0.99 | 1 | ns |
| type_person | Health & social services | Other | 292 | 19 | 0.89862907 | 20.4777545 | 0.379 | 1 | ns |
| type_person | Humanities & social science | Other | 83 | 19 | 0.8220602 | 27.9288192 | 0.418 | 1 | ns |
| type_telephone | Production | Construction | 23 | 20 | 0.778508 | 40.5844964 | 0.441 | 1 | ns |
| type_telephone | Production | Natural science | 23 | 59 | -0.13150581 | 41.8295186 | 0.896 | 1 | ns |
| type_telephone | Production | Traffic | 23 | 27 | -0.19890488 | 44.525466 | 0.843 | 1 | ns |
| type_telephone | Production | Services | 23 | 111 | 0.97459259 | 30.2060576 | 0.338 | 1 | ns |
| type_telephone | Production | Business organisation | 23 | 121 | -0.30963472 | 30.9143337 | 0.759 | 1 | ns |
| type_telephone | Production | Health & social services | 23 | 292 | -0.29334226 | 25.2474478 | 0.772 | 1 | ns |
| type_telephone | Production | Humanities & social science | 23 | 83 | -0.01620593 | 32.5729693 | 0.987 | 1 | ns |
| type_telephone | Production | Other | 23 | 19 | 0.39946758 | 39.5723712 | 0.692 | 1 | ns |
| type_telephone | Construction | Natural science | 20 | 59 | -1.04599167 | 35.4884969 | 0.303 | 1 | ns |
| type_telephone | Construction | Traffic | 20 | 27 | -1.04435427 | 39.2751931 | 0.303 | 1 | ns |
| type_telephone | Construction | Services | 20 | 111 | -0.0540316 | 25.7042145 | 0.957 | 1 | ns |
| type_telephone | Construction | Business organisation | 20 | 121 | -1.3026128 | 26.280845 | 0.204 | 1 | ns |
| type_telephone | Construction | Health & social services | 20 | 292 | -1.33689315 | 21.6497557 | 0.195 | 1 | ns |
| type_telephone | Construction | Humanities & social science | 20 | 83 | -1.0019538 | 27.6602284 | 0.325 | 1 | ns |
| type_telephone | Construction | Other | 20 | 19 | -0.38330137 | 36.9997269 | 0.704 | 1 | ns |
| type_telephone | Natural science | Traffic | 59 | 27 | -0.09568453 | 58.7156507 | 0.924 | 1 | ns |
| type_telephone | Natural science | Services | 59 | 111 | 1.56571856 | 106.154988 | 0.12 | 1 | ns |
| type_telephone | Natural science | Business organisation | 59 | 121 | -0.23042458 | 110.520746 | 0.818 | 1 | ns |
| type_telephone | Natural science | Health & social services | 59 | 292 | -0.20801966 | 78.6821281 | 0.836 | 1 | ns |
| type_telephone | Natural science | Humanities & social science | 59 | 83 | 0.17243272 | 113.185802 | 0.863 | 1 | ns |
| type_telephone | Natural science | Other | 59 | 19 | 0.60403767 | 34.5124109 | 0.55 | 1 | ns |
| type_telephone | Traffic | Services | 27 | 111 | 1.42603006 | 40.6477236 | 0.161 | 1 | ns |
| type_telephone | Traffic | Business organisation | 27 | 121 | -0.08650401 | 41.9492359 | 0.931 | 1 | ns |
| type_telephone | Traffic | Health & social services | 27 | 292 | -0.05367126 | 31.7784371 | 0.958 | 1 | ns |
| type_telephone | Traffic | Humanities & social science | 27 | 83 | 0.25252351 | 44.6926462 | 0.802 | 1 | ns |
| type_telephone | Traffic | Other | 27 | 19 | 0.63504064 | 38.278773 | 0.529 | 1 | ns |
| type_telephone | Services | Business organisation | 111 | 121 | -2.30891675 | 229.993704 | 0.022 | 0.785 | ns |
| type_telephone | Services | Health & social services | 111 | 292 | -2.73792547 | 205.069501 | 0.007 | 0.242 | ns |
| type_telephone | Services | Humanities & social science | 111 | 83 | -1.69816587 | 178.697424 | 0.091 | 1 | ns |
| type_telephone | Services | Other | 111 | 19 | -0.45128533 | 24.692753 | 0.656 | 1 | ns |
| type_telephone | Business organisation | Health & social services | 121 | 292 | 0.06663475 | 215.079937 | 0.947 | 1 | ns |
| type_telephone | Business organisation | Humanities & social science | 121 | 83 | 0.49672596 | 187.377258 | 0.62 | 1 | ns |
| type_telephone | Business organisation | Other | 121 | 19 | 0.82616278 | 25.2703237 | 0.416 | 1 | ns |
| type_telephone | Health & social services | Humanities & social science | 292 | 83 | 0.5246927 | 138.425393 | 0.601 | 1 | ns |
| type_telephone | Health & social services | Other | 292 | 19 | 0.83735944 | 20.6396929 | 0.412 | 1 | ns |
| type_telephone | Humanities & social science | Other | 83 | 19 | 0.5260215 | 26.652617 | 0.603 | 1 | ns |
| type_video | Production | Construction | 23 | 20 | 0.7626003 | 40.9994119 | 0.45 | 1 | ns |
| type_video | Production | Natural science | 23 | 59 | -0.58220728 | 35.6527316 | 0.564 | 1 | ns |
| type_video | Production | Traffic | 23 | 27 | 1.38709186 | 47.3691074 | 0.172 | 1 | ns |
| type_video | Production | Services | 23 | 111 | 0.46378889 | 29.0690294 | 0.646 | 1 | ns |
| type_video | Production | Business organisation | 23 | 121 | 0.14679178 | 29.5587503 | 0.884 | 1 | ns |
| type_video | Production | Health & social services | 23 | 292 | 0.53606731 | 25.2535396 | 0.597 | 1 | ns |
| type_video | Production | Humanities & social science | 23 | 83 | -0.64757744 | 31.88541 | 0.522 | 1 | ns |
| type_video | Production | Other | 23 | 19 | -0.07834073 | 39.9871354 | 0.938 | 1 | ns |
| type_video | Construction | Natural science | 20 | 59 | -1.58049794 | 32.7653791 | 0.124 | 1 | ns |
| type_video | Construction | Traffic | 20 | 27 | 0.66055591 | 44.3695501 | 0.512 | 1 | ns |
| type_video | Construction | Services | 20 | 111 | -0.53908508 | 26.0867568 | 0.594 | 1 | ns |
| type_video | Construction | Business organisation | 20 | 121 | -0.87079288 | 26.5822217 | 0.392 | 1 | ns |
| type_video | Construction | Health & social services | 20 | 292 | -0.5028194 | 22.2455036 | 0.62 | 1 | ns |
| type_video | Construction | Humanities & social science | 20 | 83 | -1.68827955 | 28.9394296 | 0.102 | 1 | ns |
| type_video | Construction | Other | 20 | 19 | -0.87682342 | 36.9925418 | 0.386 | 1 | ns |
| type_video | Natural science | Traffic | 59 | 27 | 2.33632019 | 42.9313185 | 0.024 | 0.871 | ns |
| type_video | Natural science | Services | 59 | 111 | 1.73735054 | 117.092574 | 0.085 | 1 | ns |
| type_video | Natural science | Business organisation | 59 | 121 | 1.21550656 | 121.703734 | 0.227 | 1 | ns |
| type_video | Natural science | Health & social services | 59 | 292 | 2.01384097 | 88.6800649 | 0.047 | 1 | ns |
| type_video | Natural science | Humanities & social science | 59 | 83 | -0.0710044 | 125.48146 | 0.944 | 1 | ns |
| type_video | Natural science | Other | 59 | 19 | 0.52004034 | 31.4525848 | 0.607 | 1 | ns |
| type_video | Traffic | Services | 27 | 111 | -1.3725527 | 34.8954817 | 0.179 | 1 | ns |
| type_video | Traffic | Business organisation | 27 | 121 | -1.69016159 | 35.5148285 | 0.1 | 1 | ns |
| type_video | Traffic | Health & social services | 27 | 292 | -1.36940208 | 30.1025826 | 0.181 | 1 | ns |
| type_video | Traffic | Humanities & social science | 27 | 83 | -2.4658583 | 38.3920031 | 0.018 | 0.655 | ns |
| type_video | Traffic | Other | 27 | 19 | -1.52450082 | 43.2303599 | 0.135 | 1 | ns |
| type_video | Services | Business organisation | 111 | 121 | -0.6059744 | 229.964687 | 0.545 | 1 | ns |
| type_video | Services | Health & social services | 111 | 292 | 0.12072631 | 219.522073 | 0.904 | 1 | ns |
| type_video | Services | Humanities & social science | 111 | 83 | -2.00853667 | 175.031087 | 0.046 | 1 | ns |
| type_video | Services | Other | 111 | 19 | -0.60422977 | 24.9057426 | 0.551 | 1 | ns |
| type_video | Business organisation | Health & social services | 121 | 292 | 0.82092481 | 230.977701 | 0.413 | 1 | ns |
| type_video | Business organisation | Humanities & social science | 121 | 83 | -1.42600418 | 182.626526 | 0.156 | 1 | ns |
| type_video | Business organisation | Other | 121 | 19 | -0.26340791 | 25.3893798 | 0.794 | 1 | ns |
| type_video | Health & social services | Humanities & social science | 292 | 83 | -2.3841927 | 142.456353 | 0.018 | 0.662 | ns |
| type_video | Health & social services | Other | 292 | 19 | -0.68967437 | 21.1581112 | 0.498 | 1 | ns |
| type_video | Humanities & social science | Other | 83 | 19 | 0.58895492 | 27.696299 | 0.561 | 1 | ns |

| *dfd = numerator degrees of freedom in the denominator ; dfn = degrees of freedom in the numerator; n = number; Production: Production of raw materials and goods, and manufacturing; Construction & architecture: Construction, architecture, surveying and technical building services; Natural science: Natural sciences, geography and informatics; Traffic: Traffic, logistics, safety and security; Services: Commercial services, trading, sales, the hotel business and tourism; Business organisation: Business organisation, accounting, law and administration; Health & Social Services: Health care, the social sector, teaching and education; Humanities & social science: Philology, literature, humanities, social sciences, economics, media, art, culture, and design; *p ≤ .05; **p < .01; ***p < .001; ns = not significant (p > .05)* |
| --- |

Table 4 Results of pairwise t-tests for dependent samples with Bonferroni correction method to compare agreement to different implementation options of type of consultation within different occupational areas

| **occupational area** | **Type of consultation** | **Type of consultation** | **n1** | **n2** | **Statistic (t)** | **df** | **p** | **p.adj** | **p.adj.signif** |
| --- | --- | --- | --- | --- | --- | --- | --- | --- | --- |
| Production | type_person | type_telephone | 23 | 23 | 6.66666667 | 22 | 0.00000106 | 0.00000318 | **** |
| Production | type_person | type_video | 23 | 23 | 5.25431662 | 22 | 0.0000285 | 0.0000855 | **** |
| Production | type_telephone | type_video | 23 | 23 | -1.49973374 | 22 | 0.148 | 0.444 | ns |
| Construction & architecture | type_person | type_telephone | 20 | 20 | 5.62731434 | 19 | 0.00002 | 0.00006 | **** |
| Construction & architecture | type_person | type_video | 20 | 20 | 5.18040306 | 19 | 0.0000532 | 0.00016 | *** |
| Construction & architecture | type_telephone | type_video | 20 | 20 | -0.97918607 | 19 | 0.34 | 1 | ns |
| Natural science | type_person | type_telephone | 59 | 59 | 8.20102677 | 58 | 2.82E-11 | 8.46E-11 | **** |
| Natural science | type_person | type_video | 59 | 59 | 6.38134238 | 58 | 3.18E-08 | 9.54E-08 | **** |
| Natural science | type_telephone | type_video | 59 | 59 | -3.12339559 | 58 | 0.003 | 0.008 | ** |
| Traffic | type_person | type_telephone | 27 | 27 | 8.76808476 | 26 | 3.04E-09 | 9.12E-09 | **** |
| Traffic | type_person | type_video | 27 | 27 | 7.63151361 | 26 | 4.23E-08 | 1.27E-07 | **** |
| Traffic | type_telephone | type_video | 27 | 27 | 0.79220339 | 26 | 0.435 | 1 | ns |
| Services | type_person | type_telephone | 111 | 111 | 15.8364783 | 110 | 3.84E-30 | 1.15E-29 | **** |
| Services | type_person | type_video | 111 | 111 | 12.3999403 | 110 | 1.27E-22 | 3.81E-22 | **** |
| Services | type_telephone | type_video | 111 | 111 | -4.4838937 | 110 | 0.0000181 | 0.0000543 | **** |
| Business organisation | type_person | type_telephone | 121 | 121 | 11.9938536 | 120 | 2.88E-22 | 8.64E-22 | **** |
| Business organisation | type_person | type_video | 121 | 121 | 10.651074 | 120 | 4.72E-19 | 1.42E-18 | **** |
| Business organisation | type_telephone | type_video | 121 | 121 | -1.78498582 | 120 | 0.077 | 0.23 | ns |
| Health & social services | type_person | type_telephone | 292 | 292 | 21.1947105 | 291 | 6.05E-61 | 1.82E-60 | **** |
| Health & social services | type_person | type_video | 292 | 292 | 19.8012307 | 291 | 7.4E-56 | 2.22E-55 | **** |
| Health & social services | type_telephone | type_video | 292 | 292 | -1.59374021 | 291 | 0.112 | 0.336 | ns |
| Humanities & social science | type_person | type_telephone | 83 | 83 | 13.6406324 | 82 | 8.49E-23 | 2.55E-22 | **** |
| Humanities & social science | type_person | type_video | 83 | 83 | 9.01176146 | 82 | 6.82E-14 | 2.05E-13 | **** |
| Humanities & social science | type_telephone | type_video | 83 | 83 | -4.00697999 | 82 | 0.000135 | 0.000405 | *** |
| Other | type_person | type_telephone | 19 | 19 | 5.92862958 | 18 | 0.000013 | 0.000039 | **** |
| Other | type_person | type_video | 19 | 19 | 3.91050322 | 18 | 0.001 | 0.003 | ** |
| Other | type_telephone | type_video | 19 | 19 | -2.37915476 | 18 | 0.029 | 0.086 | ns |

| *dfd = numerator degrees of freedom in the denominator ; dfn = degrees of freedom in the numerator; n = number; Production: Production of raw materials and goods, and manufacturing; Construction & architecture: Construction, architecture, surveying and technical building services; Natural science: Natural sciences, geography and informatics; Traffic: Traffic, logistics, safety and security; Services: Commercial services, trading, sales, the hotel business and tourism; Business organisation: Business organisation, accounting, law and administration; Health & Social Services: Health care, the social sector, teaching and education; Humanities & social science: Philology, literature, humanities, social sciences, economics, media, art, culture, and design; *p ≤ .05; **p < .01; ***p < .001; ns = not significant (p > .05)* |
| --- |

Table 5 Results of pairwise t-tests for independent samples with Bonferroni correction method to analyse differences of agreement to different implementation options of location of consultation at work between different occupational areas (n=755)

| **Location of consultation** | **Occupational area** | **Occupational area** | **n1** | **n2** | **Statistic (t)** | **df** | **p** | **p.adj** | **p.adj.signif** |
| --- | --- | --- | --- | --- | --- | --- | --- | --- | --- |
| location_extern | Production | Construction | 23 | 20 | -0.52490222 | 40.4825831 | 0.603 | 1 | ns |
| location_extern | Production | Natural sciences | 23 | 59 | -0.87247625 | 30.2179832 | 0.39 | 1 | ns |
| location_extern | Production | Traffic | 23 | 27 | -1.18309403 | 39.6983898 | 0.244 | 1 | ns |
| location_extern | Production | Services | 23 | 111 | -1.06522076 | 25.4806986 | 0.297 | 1 | ns |
| location_extern | Production | Business organisation | 23 | 121 | -0.0316115 | 28.6506501 | 0.975 | 1 | ns |
| location_extern | Production | Health & social services | 23 | 292 | 0.00841619 | 24.7780564 | 0.993 | 1 | ns |
| location_extern | Production | Humanities & social science | 23 | 83 | -1.13917349 | 26.5133866 | 0.265 | 1 | ns |
| location_extern | Production | Other | 23 | 19 | -2.06625831 | 28.2466159 | 0.048 | 1 | ns |
| location_extern | Construction | Natural sciences | 20 | 59 | -0.2856255 | 29.5483416 | 0.777 | 1 | ns |
| location_extern | Construction | Traffic | 20 | 27 | -0.69807424 | 39.8305944 | 0.489 | 1 | ns |
| location_extern | Construction | Services | 20 | 111 | -0.48500542 | 23.4408918 | 0.632 | 1 | ns |
| location_extern | Construction | Business organisation | 20 | 121 | 0.71120606 | 27.5724523 | 0.483 | 1 | ns |
| location_extern | Construction | Health & social services | 20 | 292 | 0.79825826 | 22.5399093 | 0.433 | 1 | ns |
| location_extern | Construction | Humanities & social science | 20 | 83 | -0.57841999 | 24.7710647 | 0.568 | 1 | ns |
| location_extern | Construction | Other | 20 | 19 | -1.67736117 | 26.7733601 | 0.105 | 1 | ns |
| location_extern | Natural sciences | Traffic | 59 | 27 | -0.60274287 | 47.1115673 | 0.55 | 1 | ns |
| location_extern | Natural sciences | Services | 59 | 111 | -0.31232172 | 107.96691 | 0.755 | 1 | ns |
| location_extern | Natural sciences | Business organisation | 59 | 121 | 1.60892788 | 143.54805 | 0.11 | 1 | ns |
| location_extern | Natural sciences | Health & social services | 59 | 292 | 1.94950122 | 102.202699 | 0.054 | 1 | ns |
| location_extern | Natural sciences | Humanities & social science | 59 | 83 | -0.46813886 | 115.093962 | 0.641 | 1 | ns |
| location_extern | Natural sciences | Other | 59 | 19 | -2.21410163 | 60.4069315 | 0.031 | 1 | ns |
| location_extern | Traffic | Services | 27 | 111 | 0.44172453 | 35.2355824 | 0.661 | 1 | ns |
| location_extern | Traffic | Business organisation | 27 | 121 | 1.79920521 | 43.9163727 | 0.079 | 1 | ns |
| location_extern | Traffic | Health & social services | 27 | 292 | 1.9950323 | 33.3977798 | 0.054 | 1 | ns |
| location_extern | Traffic | Humanities & social science | 27 | 83 | 0.31347727 | 37.942211 | 0.756 | 1 | ns |
| location_extern | Traffic | Other | 27 | 19 | -1.00988835 | 39.5790904 | 0.319 | 1 | ns |
| location_extern | Services | Business organisation | 111 | 121 | 2.28374865 | 215.564888 | 0.023 | 0.842 | ns |
| location_extern | Services | Health & social services | 111 | 292 | 2.99622296 | 286.837905 | 0.003 | 0.107 | ns |
| location_extern | Services | Humanities & social science | 111 | 83 | -0.2120393 | 178.406621 | 0.832 | 1 | ns |
| location_extern | Services | Other | 111 | 19 | -2.33705905 | 39.7517216 | 0.025 | 0.886 | ns |
| location_extern | Business organisation | Health & social services | 121 | 292 | 0.09386335 | 227.628818 | 0.925 | 1 | ns |
| location_extern | Business organisation | Humanities & social science | 121 | 83 | -2.3563777 | 201.991494 | 0.019 | 0.698 | ns |
| location_extern | Business organisation | Other | 121 | 19 | -4.03791574 | 61.0771569 | 0.000153 | 0.006 | ** |
| location_extern | Health & social services | Humanities & social science | 292 | 83 | -3.00143291 | 193.954295 | 0.003 | 0.109 | ns |
| location_extern | Health & social services | Other | 292 | 19 | -4.85471646 | 35.6275285 | 0.0000239 | 0.00086 | *** |
| location_extern | Humanities & social science | Other | 83 | 19 | -2.04988692 | 45.6788912 | 0.046 | 1 | ns |
| location_intern | Production | Construction | 23 | 20 | 1.64834771 | 40.9991349 | 0.107 | 1 | ns |
| location_intern | Production | Natural sciences | 23 | 59 | 0.96631716 | 35.9745759 | 0.34 | 1 | ns |
| location_intern | Production | Traffic | 23 | 27 | 1.22201912 | 43.6123274 | 0.228 | 1 | ns |
| location_intern | Production | Services | 23 | 111 | 1.16438061 | 29.9772231 | 0.253 | 1 | ns |
| location_intern | Production | Business organisation | 23 | 121 | 0.10812605 | 30.3852958 | 0.915 | 1 | ns |
| location_intern | Production | Health & social services | 23 | 292 | 0.6582856 | 24.968979 | 0.516 | 1 | ns |
| location_intern | Production | Humanities & social science | 23 | 83 | 1.614047 | 30.2769846 | 0.117 | 1 | ns |
| location_intern | Production | Other | 23 | 19 | 1.38213317 | 38.6765643 | 0.175 | 1 | ns |
| location_intern | Construction | Natural sciences | 20 | 59 | -1.06508584 | 33.0696218 | 0.295 | 1 | ns |
| location_intern | Construction | Traffic | 20 | 27 | -0.57149434 | 40.6686131 | 0.571 | 1 | ns |
| location_intern | Construction | Services | 20 | 111 | -0.97479711 | 26.9919326 | 0.338 | 1 | ns |
| location_intern | Construction | Business organisation | 20 | 121 | -2.08587457 | 27.4047838 | 0.046 | 1 | ns |
| location_intern | Construction | Health & social services | 20 | 292 | -1.62753703 | 21.9554731 | 0.118 | 1 | ns |
| location_intern | Construction | Humanities & social science | 20 | 83 | -0.49109932 | 27.2942122 | 0.627 | 1 | ns |
| location_intern | Construction | Other | 20 | 19 | -0.14837825 | 35.8530196 | 0.883 | 1 | ns |
| location_intern | Natural sciences | Traffic | 59 | 27 | 0.47317494 | 51.7556289 | 0.638 | 1 | ns |
| location_intern | Natural sciences | Services | 59 | 111 | 0.23000792 | 121.880967 | 0.818 | 1 | ns |
| location_intern | Natural sciences | Business organisation | 59 | 121 | -1.42242278 | 125.956414 | 0.157 | 1 | ns |
| location_intern | Natural sciences | Health & social services | 59 | 292 | -0.69832608 | 85.2431964 | 0.487 | 1 | ns |
| location_intern | Natural sciences | Humanities & social science | 59 | 83 | 0.93949396 | 117.85184 | 0.349 | 1 | ns |
| location_intern | Natural sciences | Other | 59 | 19 | 0.77194734 | 27.7444359 | 0.447 | 1 | ns |
| location_intern | Traffic | Services | 27 | 111 | -0.33313533 | 41.4801686 | 0.741 | 1 | ns |
| location_intern | Traffic | Business organisation | 27 | 121 | -1.60794987 | 42.3134657 | 0.115 | 1 | ns |
| location_intern | Traffic | Health & social services | 27 | 292 | -1.05740963 | 31.7303579 | 0.298 | 1 | ns |
| location_intern | Traffic | Humanities & social science | 27 | 83 | 0.2183667 | 41.9214105 | 0.828 | 1 | ns |
| location_intern | Traffic | Other | 27 | 19 | 0.36151797 | 35.1749234 | 0.72 | 1 | ns |
| location_intern | Services | Business organisation | 111 | 121 | -1.93288193 | 229.901235 | 0.054 | 1 | ns |
| location_intern | Services | Health & social services | 111 | 292 | -1.19406472 | 199.528675 | 0.234 | 1 | ns |
| location_intern | Services | Humanities & social science | 111 | 83 | 0.83313434 | 186.676305 | 0.406 | 1 | ns |
| location_intern | Services | Other | 111 | 19 | 0.67241562 | 23.4867822 | 0.508 | 1 | ns |
| location_intern | Business organisation | Health & social services | 121 | 292 | 1.13757696 | 212.370168 | 0.257 | 1 | ns |
| location_intern | Business organisation | Humanities & social science | 121 | 83 | 2.73650516 | 194.963764 | 0.007 | 0.244 | ns |
| location_intern | Business organisation | Other | 121 | 19 | 1.64931198 | 23.7632029 | 0.112 | 1 | ns |
| location_intern | Health & social services | Humanities & social science | 292 | 83 | 2.17266931 | 147.785547 | 0.031 | 1 | ns |
| location_intern | Health & social services | Other | 292 | 19 | 1.21942632 | 20.0441451 | 0.237 | 1 | ns |
| location_intern | Humanities & social science | Other | 83 | 19 | 0.24890926 | 23.7073769 | 0.806 | 1 | ns |

| *dfd = numerator degrees of freedom in the denominator ; dfn = degrees of freedom in the numerator; n = number; Production: Production of raw materials and goods, and manufacturing; Construction & architecture: Construction, architecture, surveying and technical building services; Natural science: Natural sciences, geography and informatics; Traffic: Traffic, logistics, safety and security; Services: Commercial services, trading, sales, the hotel business and tourism; Business organisation: Business organisation, accounting, law and administration; Health & Social Services: Health care, the social sector, teaching and education; Humanities & social science: Philology, literature, humanities, social sciences, economics, media, art, culture, and design; *p ≤ .05; **p < .01; ***p < .001; ns = not significant (p > .05)* |
| --- |

Table 6 Results of pairwise t-tests for dependent samples with Bonferroni correction method to compare agreement to different implementation options of location of consultation within different occupational areas

| **occupational area** | **Location of consultation** | **Location of consultation** | **n1** | **n2** | **Statistic (t)** | **df** | **p** | **p.adj** | **p.adj.signif** |
| --- | --- | --- | --- | --- | --- | --- | --- | --- | --- |
| Production | location_extern | location_intern | 23 | 23 | 3.7714339 | 22 | 0.001 | 0.001 | ** |
| Construction & architecture | location_extern | location_intern | 20 | 20 | 6.32801902 | 19 | 0.00000451 | 0.00000451 | **** |
| Natural science | location_extern | location_intern | 59 | 59 | 9.53922942 | 58 | 1.73E-13 | 1.73E-13 | **** |
| Traffic | location_extern | location_intern | 27 | 27 | 6.72686417 | 26 | 3.88E-07 | 3.88E-07 | **** |
| Services | location_extern | location_intern | 111 | 111 | 13.5758289 | 110 | 2.97E-25 | 2.97E-25 | **** |
| Business organisation | location_extern | location_intern | 121 | 121 | 8.54167875 | 120 | 4.9E-14 | 4.9E-14 | **** |
| Health & social services | location_extern | location_intern | 292 | 292 | 14.8589682 | 291 | 1.49E-37 | 1.49E-37 | **** |
| Humanities & social science | location_extern | location_intern | 83 | 83 | 14.1592649 | 82 | 9.81E-24 | 9.81E-24 | **** |
| Other | location_extern | location_intern | 19 | 19 | 7.13895109 | 18 | 0.00000119 | 0.00000119 | **** |

| *dfd = numerator degrees of freedom in the denominator ; dfn = degrees of freedom in the numerator; n = number; Production: Production of raw materials and goods, and manufacturing; Construction & architecture: Construction, architecture, surveying and technical building services; Natural science: Natural sciences, geography and informatics; Traffic: Traffic, logistics, safety and security; Services: Commercial services, trading, sales, the hotel business and tourism; Business organisation: Business organisation, accounting, law and administration; Health & Social Services: Health care, the social sector, teaching and education; Humanities & social science: Philology, literature, humanities, social sciences, economics, media, art, culture, and design;* **p ≤ .05; **p < .01; ***p < .001; ns = not significant (p > .05)* |
| --- |

**Company size**

Table 7 Results of pairwise t-tests for independent samples with Bonferroni correction method to analyse differences of agreement to different implementation options of location of consultation at work between different company sizes (n=755)

| **Location of consultation** | **Company size*** | **Company size*** | **n1** | **n2** | **Statistic (t)** | **df** | **p** | **p.adj** | **p.adj.signif** |
| --- | --- | --- | --- | --- | --- | --- | --- | --- | --- |
| location_extern | "1-9" | "10-49" | 87 | 157 | -0.35887698 | 165.816309 | 0.72 | 1 | ns |
| location_extern | "1-9" | "50-249" | 87 | 161 | 0.52130404 | 187.960635 | 0.603 | 1 | ns |
| location_extern | "1-9" | "250-999" | 87 | 134 | 0.60636556 | 190.8897 | 0.545 | 1 | ns |
| location_extern | "1-9" | "≥ 1000" | 87 | 216 | 0.22847818 | 155.792764 | 0.82 | 1 | ns |
| location_extern | "10-49" | "50-249" | 157 | 161 | 1.04990528 | 310.785972 | 0.295 | 1 | ns |
| location_extern | "10-49" | "250-999" | 157 | 134 | 1.12651251 | 266.28008 | 0.261 | 1 | ns |
| location_extern | "10-49" | "≥ 1000" | 157 | 216 | 0.76095705 | 347.092982 | 0.447 | 1 | ns |
| location_extern | "50-249" | "250-999" | 161 | 134 | 0.11283746 | 285.287592 | 0.91 | 1 | ns |
| location_extern | "50-249" | "≥ 1000" | 161 | 216 | -0.39360767 | 325.551475 | 0.694 | 1 | ns |
| location_extern | "250-999" | "≥ 1000" | 134 | 216 | -0.50059269 | 265.709908 | 0.617 | 1 | ns |
| location_intern | "1-9" | "10-49" | 87 | 157 | 0.48346519 | 175.772261 | 0.629 | 1 | ns |
| location_intern | "1-9" | "50-249" | 87 | 161 | -1.57692863 | 184.061189 | 0.117 | 1 | ns |
| location_intern | "1-9" | "250-999" | 87 | 134 | -1.53217888 | 193.201664 | 0.127 | 1 | ns |
| location_intern | "1-9" | "≥ 1000" | 87 | 216 | -2.64713231 | 174.440786 | 0.009 | 0.089 | ns |
| location_intern | "10-49" | "50-249" | 157 | 161 | -2.43120152 | 315.568045 | 0.016 | 0.156 | ns |
| location_intern | "10-49" | "250-999" | 157 | 134 | -2.31215544 | 272.847369 | 0.021 | 0.215 | ns |
| location_intern | "10-49" | "≥ 1000" | 157 | 216 | -3.7731939 | 355.443086 | 0.000189 | 0.002 | ** |
| location_intern | "50-249" | "250-999" | 161 | 134 | -0.02983212 | 280.932315 | 0.976 | 1 | ns |
| location_intern | "50-249" | "≥ 1000" | 161 | 216 | -1.18863444 | 353.557293 | 0.235 | 1 | ns |
| location_intern | "250-999" | "≥ 1000" | 134 | 216 | -1.0814744 | 287.798124 | 0.28 | 1 | ns |

**given as number of employees; df=degrees of freedom, n = number; *p ≤ .05; **p < .01; ***p < .001; ns = not significant (p > .05)*

Table 8 Results of pairwise t-tests for dependent samples with Bonferroni correction method to compare agreement to different implementation options of location of consultation within different company sizes

| **Company size*** | **Location of consultation** | **Location of consultation** | **n1** | **n2** | **Statistic (t)** | **df** | **p** | **p.adj** | **p.adj.signif** |
| --- | --- | --- | --- | --- | --- | --- | --- | --- | --- |
| "1-9" | location_extern | location_intern | 87 | 87 | 10.6762837 | 86 | 1.96E-17 | 1.96E-17 | **** |
| "10-49" | location_extern | location_intern | 157 | 157 | 16.718132 | 156 | 1.33E-36 | 1.33E-36 | **** |
| "50-249" | location_extern | location_intern | 161 | 161 | 12.3121351 | 160 | 6.25E-25 | 6.25E-25 | **** |
| "250-999" | location_extern | location_intern | 134 | 134 | 10.9056257 | 133 | 3.54E-20 | 3.54E-20 | **** |
| "≥ 1000" | location_extern | location_intern | 216 | 216 | 13.0591327 | 215 | 4.41E-29 | 4.41E-29 | **** |

**given as number of employees; df=degrees of freedom, n = number; *p ≤ .05; **p < .01; ***p < .001; ns = not significant (p > .05)*

Table 9 Results of pairwise t-tests for independent samples with Bonferroni correction method to analyse differences of agreement to different implementation options of purpose of consultation at work between different company sizes (n=755)

| **Purpose of consultation** | **Company size*** | **Company size*** | **n1** | **n2** | **Statistic (t)** | **df** | **p** | **p.adj** | **p.adj.signif** |
| --- | --- | --- | --- | --- | --- | --- | --- | --- | --- |
| purpose_occupational | "1-9" | "10-49" | 87 | 157 | -0.2432989 | 160.939017 | 0.808 | 1 | ns |
| purpose_occupational | "1-9" | "50-249" | 87 | 161 | -1.54461804 | 143.357252 | 0.125 | 1 | ns |
| purpose_occupational | "1-9" | "250-999" | 87 | 134 | -1.78654256 | 139.05644 | 0.076 | 0.762 | ns |
| purpose_occupational | "1-9" | "≥ 1000" | 87 | 216 | -2.0999085 | 119.832841 | 0.038 | 0.378 | ns |
| purpose_occupational | "10-49" | "50-249" | 157 | 161 | -1.69957002 | 308.372096 | 0.09 | 0.902 | ns |
| purpose_occupational | "10-49" | "250-999" | 157 | 134 | -2.02554847 | 286.594946 | 0.044 | 0.437 | ns |
| purpose_occupational | "10-49" | "≥ 1000" | 157 | 216 | -2.52184058 | 281.353415 | 0.012 | 0.122 | ns |
| purpose_occupational | "50-249" | "250-999" | 161 | 134 | -0.33211433 | 291.749565 | 0.74 | 1 | ns |
| purpose_occupational | "50-249" | "≥ 1000" | 161 | 216 | -0.70903672 | 318.68636 | 0.479 | 1 | ns |
| purpose_occupational | "250-999" | "≥ 1000" | 134 | 216 | -0.34563907 | 279.848452 | 0.73 | 1 | ns |
| purpose_maintain | "1-9" | "10-49" | 87 | 157 | -0.65204564 | 173.547894 | 0.515 | 1 | ns |
| purpose_maintain | "1-9" | "50-249" | 87 | 161 | -1.21241476 | 165.414646 | 0.227 | 1 | ns |
| purpose_maintain | "1-9" | "250-999" | 87 | 134 | -1.5518326 | 147.212005 | 0.123 | 1 | ns |
| purpose_maintain | "1-9" | "≥ 1000" | 87 | 216 | -1.71026818 | 135.966118 | 0.09 | 0.895 | ns |
| purpose_maintain | "10-49" | "50-249" | 157 | 161 | -0.66597392 | 314.353936 | 0.506 | 1 | ns |
| purpose_maintain | "10-49" | "250-999" | 157 | 134 | -1.06855858 | 285.97836 | 0.286 | 1 | ns |
| purpose_maintain | "10-49" | "≥ 1000" | 157 | 216 | -1.25950766 | 302.764993 | 0.209 | 1 | ns |
| purpose_maintain | "50-249" | "250-999" | 161 | 134 | -0.37799069 | 292.706232 | 0.706 | 1 | ns |
| purpose_maintain | "50-249" | "≥ 1000" | 161 | 216 | -0.53942333 | 321.994998 | 0.59 | 1 | ns |
| purpose_maintain | "250-999" | "≥ 1000" | 134 | 216 | -0.15081187 | 303.762973 | 0.88 | 1 | ns |
| purpose_private | "1-9" | "10-49" | 87 | 157 | 1.13781143 | 196.405326 | 0.257 | 1 | ns |
| purpose_private | "1-9" | "50-249" | 87 | 161 | -0.23114233 | 167.908847 | 0.817 | 1 | ns |
| purpose_private | "1-9" | "250-999" | 87 | 134 | 0.37089274 | 186.62492 | 0.711 | 1 | ns |
| purpose_private | "1-9" | "≥ 1000" | 87 | 216 | 2.02681461 | 165.15833 | 0.044 | 0.443 | ns |
| purpose_private | "10-49" | "50-249" | 157 | 161 | -1.62152675 | 303.653832 | 0.106 | 1 | ns |
| purpose_private | "10-49" | "250-999" | 157 | 134 | -0.85507706 | 288.002432 | 0.393 | 1 | ns |
| purpose_private | "10-49" | "≥ 1000" | 157 | 216 | 0.88248192 | 320.104283 | 0.378 | 1 | ns |
| purpose_private | "50-249" | "250-999" | 161 | 134 | 0.70742445 | 274.181992 | 0.48 | 1 | ns |
| purpose_private | "50-249" | "≥ 1000" | 161 | 216 | 2.82739764 | 361.04688 | 0.005 | 0.05 | * |
| purpose_private | "250-999" | "≥ 1000" | 134 | 216 | 1.84631411 | 286.468278 | 0.066 | 0.659 | ns |
| purpose_reintegration | "1-9" | "10-49" | 87 | 157 | -0.26320127 | 177.663508 | 0.793 | 1 | ns |
| purpose_reintegration | "1-9" | "50-249" | 87 | 161 | 0.00479714 | 183.135593 | 0.996 | 1 | ns |
| purpose_reintegration | "1-9" | "250-999" | 87 | 134 | -0.25050995 | 174.411233 | 0.802 | 1 | ns |
| purpose_reintegration | "1-9" | "≥ 1000" | 87 | 216 | -1.78091072 | 128.26077 | 0.077 | 0.773 | ns |
| purpose_reintegration | "10-49" | "50-249" | 157 | 161 | 0.31242035 | 315.891941 | 0.755 | 1 | ns |
| purpose_reintegration | "10-49" | "250-999" | 157 | 134 | 0.01409442 | 286.775083 | 0.989 | 1 | ns |
| purpose_reintegration | "10-49" | "≥ 1000" | 157 | 216 | -1.85850727 | 279.655214 | 0.064 | 0.641 | ns |
| purpose_reintegration | "50-249" | "250-999" | 161 | 134 | -0.29729892 | 291.591311 | 0.766 | 1 | ns |
| purpose_reintegration | "50-249" | "≥ 1000" | 161 | 216 | -2.18592531 | 279.652516 | 0.03 | 0.297 | ns |
| purpose_reintegration | "250-999" | "≥ 1000" | 134 | 216 | -1.86446136 | 240.969169 | 0.064 | 0.635 | ns |

**given as number of employees; df=degrees of freedom, n = number; *p ≤ .05; **p < .01; ***p < .001; ns = not significant (p > .05)*

Table 10 Results of pairwise t-tests for dependent samples with Bonferroni correction method to compare agreement to different implementation options of purpose of consultation within different company sizes

| **Company size** | **Purpose of consultation** | **Purpose of consultation** | **n1** | **n2** | **Statistic (t)** | **df** | **p** | **p.adj** | **p.adj.signif** |
| --- | --- | --- | --- | --- | --- | --- | --- | --- | --- |
| "1-9" | purpose_occupational | purpose_maintain | 87 | 87 | -0.31263582 | 86 | 0.755 | 1 | ns |
| "1-9" | purpose_occupational | purpose_private | 87 | 87 | 0.82529394 | 86 | 0.411 | 1 | ns |
| "1-9" | purpose_occupational | purpose_reintegration | 87 | 87 | -0.14823092 | 86 | 0.883 | 1 | ns |
| "1-9" | purpose_maintain | purpose_private | 87 | 87 | 1.2676261 | 86 | 0.208 | 1 | ns |
| "1-9" | purpose_maintain | purpose_reintegration | 87 | 87 | 0.21701954 | 86 | 0.829 | 1 | ns |
| "1-9" | purpose_private | purpose_reintegration | 87 | 87 | -0.93508467 | 86 | 0.352 | 1 | ns |
| "10-49" | purpose_occupational | purpose_maintain | 157 | 157 | -1.16911181 | 156 | 0.244 | 1 | ns |
| "10-49" | purpose_occupational | purpose_private | 157 | 157 | 2.79666768 | 156 | 0.006 | 0.035 | * |
| "10-49" | purpose_occupational | purpose_reintegration | 157 | 157 | -0.26412051 | 156 | 0.792 | 1 | ns |
| "10-49" | purpose_maintain | purpose_private | 157 | 157 | 4.3169208 | 156 | 0.000028 | 0.000168 | *** |
| "10-49" | purpose_maintain | purpose_reintegration | 157 | 157 | 0.77105103 | 156 | 0.442 | 1 | ns |
| "10-49" | purpose_private | purpose_reintegration | 157 | 157 | -2.89058227 | 156 | 0.004 | 0.026 | * |
| "50-249" | purpose_occupational | purpose_maintain | 161 | 161 | 0.2450511 | 160 | 0.807 | 1 | ns |
| "50-249" | purpose_occupational | purpose_private | 161 | 161 | 3.24916568 | 160 | 0.001 | 0.008 | ** |
| "50-249" | purpose_occupational | purpose_reintegration | 161 | 161 | 2.09599618 | 160 | 0.038 | 0.226 | ns |
| "50-249" | purpose_maintain | purpose_private | 161 | 161 | 3.13017592 | 160 | 0.002 | 0.012 | * |
| "50-249" | purpose_maintain | purpose_reintegration | 161 | 161 | 2.30156918 | 160 | 0.023 | 0.136 | ns |
| "50-249" | purpose_private | purpose_reintegration | 161 | 161 | -1.04146728 | 160 | 0.299 | 1 | ns |
| "250-999" | purpose_occupational | purpose_maintain | 134 | 134 | 0.17477592 | 133 | 0.862 | 1 | ns |
| "250-999" | purpose_occupational | purpose_private | 134 | 134 | 4.07677071 | 133 | 0.0000781 | 0.000469 | *** |
| "250-999" | purpose_occupational | purpose_reintegration | 134 | 134 | 2.19740106 | 133 | 0.03 | 0.178 | ns |
| "250-999" | purpose_maintain | purpose_private | 134 | 134 | 4.45830431 | 133 | 0.0000174 | 0.000104 | *** |
| "250-999" | purpose_maintain | purpose_reintegration | 134 | 134 | 1.9039992 | 133 | 0.059 | 0.355 | ns |
| "250-999" | purpose_private | purpose_reintegration | 134 | 134 | -2.19808087 | 133 | 0.03 | 0.178 | ns |
| "≥ 1000" | purpose_occupational | purpose_maintain | 216 | 216 | 0.54406047 | 215 | 0.587 | 1 | ns |
| "≥ 1000" | purpose_occupational | purpose_private | 216 | 216 | 8.15146515 | 215 | 2.95E-14 | 1.77E-13 | **** |
| "≥ 1000" | purpose_occupational | purpose_reintegration | 216 | 216 | 0.44150078 | 215 | 0.659 | 1 | ns |
| "≥ 1000" | purpose_maintain | purpose_private | 216 | 216 | 7.52583505 | 215 | 1.42E-12 | 8.52E-12 | **** |
| "≥ 1000" | purpose_maintain | purpose_reintegration | 216 | 216 | -0.08784262 | 215 | 0.93 | 1 | ns |
| "≥ 1000" | purpose_private | purpose_reintegration | 216 | 216 | -7.26866566 | 215 | 6.63E-12 | 3.98E-11 | **** |

**given as number of employees; df=degrees of freedom, n = number; *p ≤ .05; **p < .01; ***p < .001; ns = not significant (p > .05)*

**Supervisor function**

Table 11 Results of pairwise t-tests for independent samples with Bonferroni correction method to analyse differences of agreement to different implementation options of location of consultation at work between employees with and without supervisor function (n=755)

| **Location of consultation** | **Supervisor function** | **Supervisor function** | **n1** | **n2** | **Statistic (t)** | **df** | **p** | **p.adj** | **p.adj.signif** |
| --- | --- | --- | --- | --- | --- | --- | --- | --- | --- |
| location_extern | without | with | 603 | 152 | -0.60224089 | 250.778454 | 0.548 | 0.548 | ns |
| location_intern | without | with | 603 | 152 | 2.52397174 | 232.885537 | 0.012 | 0.012 | * |

Table 12 Results of pairwise t-tests for dependent samples with Bonferroni correction method to compare agreement to different implementation options of location of consultation within different company sizes

| **Supervisor function** | **Location of consultation** | **Location of consultation** | **n1** | **n2** | **Statistic (t)** | **df** | **p** | **p.adj** | **p.adj.signif** |
| --- | --- | --- | --- | --- | --- | --- | --- | --- | --- |
| without | location_extern | location_intern | 603 | 603 | 24.3022257 | 602 | 1.98E-91 | 1.98E-91 | **** |
| with | location_extern | location_intern | 152 | 152 | 14.5093358 | 151 | 2E-30 | 2E-30 | **** |

*df=degrees of freedom, n = number; *p ≤ .05; **p < .01; ***p < .001; ns = not significant (p > .05)*

**Requirement level**

Table 13 Results of pairwise t-tests for independent samples with Bonferroni correction method to analyse differences of agreement to different implementation options of type of consultation at work between different requirement levels (n=7

| **Type of consultation** | **Requirement level** | **Requirement level** | **n1** | **n2** | **Statistic (t)** | **df** | **p** | **p.adj** | **p.adj.signif** |
| --- | --- | --- | --- | --- | --- | --- | --- | --- | --- |
| type_person | unskilled or semiskilled activities | specialist activities | 95 | 246 | 1.84040541 | 296.033403 | 0.067 | 0.4 | ns |
| type_person | unskilled or semiskilled activities | complex specialist activities | 95 | 239 | 2.46429245 | 307.562442 | 0.014 | 0.086 | ns |
| type_person | unskilled or semiskilled activities | highly complex activities | 95 | 175 | 2.13828346 | 267.971384 | 0.033 | 0.2 | ns |
| type_person | specialist activities | complex specialist activities | 246 | 239 | 0.67171332 | 479.001981 | 0.502 | 1 | ns |
| type_person | specialist activities | highly complex activities | 246 | 175 | 0.47869322 | 369.798672 | 0.632 | 1 | ns |
| type_person | complex specialist activities | highly complex activities | 239 | 175 | -0.14566744 | 383.323785 | 0.884 | 1 | ns |
| type_telephone | unskilled or semiskilled activities | specialist activities | 95 | 246 | -1.47030109 | 158.84695 | 0.143 | 0.858 | ns |
| type_telephone | unskilled or semiskilled activities | complex specialist activities | 95 | 239 | -1.8754609 | 163.664728 | 0.062 | 0.375 | ns |
| type_telephone | unskilled or semiskilled activities | highly complex activities | 95 | 175 | -2.17552159 | 188.900613 | 0.031 | 0.185 | ns |
| type_telephone | specialist activities | complex specialist activities | 246 | 239 | -0.5915435 | 481.722555 | 0.554 | 1 | ns |
| type_telephone | specialist activities | highly complex activities | 246 | 175 | -1.07381686 | 361.016505 | 0.284 | 1 | ns |
| type_telephone | complex specialist activities | highly complex activities | 239 | 175 | -0.53541751 | 367.10871 | 0.593 | 1 | ns |
| type_video | unskilled or semiskilled activities | specialist activities | 95 | 246 | -0.09993315 | 163.21274 | 0.921 | 1 | ns |
| type_video | unskilled or semiskilled activities | complex specialist activities | 95 | 239 | -1.13187815 | 170.076432 | 0.259 | 1 | ns |
| type_video | unskilled or semiskilled activities | highly complex activities | 95 | 175 | -2.89608355 | 178.652976 | 0.004 | 0.026 | * |
| type_video | specialist activities | complex specialist activities | 246 | 239 | -1.42095079 | 481.071584 | 0.156 | 0.936 | ns |
| type_video | specialist activities | highly complex activities | 246 | 175 | -3.77103123 | 383.328287 | 0.000188 | 0.001 | ** |
| type_video | complex specialist activities | highly complex activities | 239 | 175 | -2.36094513 | 389.353009 | 0.019 | 0.112 | ns |

*df=degrees of freedom, n = number; *p ≤ .05; **p < .01; ***p < .001; ns = not significant (p > .05)*

Table 14 Results of pairwise t-tests for dependent samples with Bonferroni correction method to compare agreement to different implementation options of type of consultation within different requirement levels

| **Requirement level_Beruf** | **Type of consultation** | **Type of consulation** | **n1** | **n2** | **Statistic (t)** | **df** | **p** | **p.adj** | **p.adj.signif** |
| --- | --- | --- | --- | --- | --- | --- | --- | --- | --- |
| unskilled or semiskilled activities | type_person | type_telephone | 95 | 95 | 15.506533 | 94 | 1.2E-27 | 3.6E-27 | **** |
| unskilled or semiskilled activities | type_person | type_video | 95 | 95 | 12.7227776 | 94 | 3.73E-22 | 1.12E-21 | **** |
| unskilled or semiskilled activities | type_telephone | type_video | 95 | 95 | -2.75918813 | 94 | 0.007 | 0.021 | * |
| specialist activities | type_person | type_telephone | 246 | 246 | 20.4554313 | 245 | 6.45E-55 | 1.93E-54 | **** |
| specialist activities | type_person | type_video | 246 | 246 | 18.9862983 | 245 | 4.83E-50 | 1.45E-49 | **** |
| specialist activities | type_telephone | type_video | 246 | 246 | -1.7507773 | 245 | 0.081 | 0.244 | ns |
| complex specialist activities | type_person | type_telephone | 239 | 239 | 18.6849501 | 238 | 1.44E-48 | 4.32E-48 | **** |
| complex specialist activities | type_person | type_video | 239 | 239 | 16.1324803 | 238 | 4.66E-40 | 1.4E-39 | **** |
| complex specialist activities | type_telephone | type_video | 239 | 239 | -2.91253199 | 238 | 0.004 | 0.012 | * |
| highly complex activities | type_person | type_telephone | 175 | 175 | 15.8175569 | 174 | 1.67E-35 | 5.01E-35 | **** |
| highly complex activities | type_person | type_video | 175 | 175 | 11.6076316 | 174 | 1.96E-23 | 5.88E-23 | **** |
| highly complex activities | type_telephone | type_video | 175 | 175 | -4.97184079 | 174 | 0.00000158 | 0.00000474 | **** |

*df=degrees of freedom, n = number; *p ≤ .05; **p < .01; ***p < .001; ns = not significant (p > .05)*
