# Supplementary material for: Employees’ preferences on organisational aspects of psychotherapeutic consultation at work by occupational area, company size, requirement levels and supervisor function – a cross-sectional study in Germany
Source: BMC Public Health. 2023 Feb 16;23:347. doi: 10.1186/s12889-023-15255-0 (PMC9932407; doi:10.1186/s12889-023-15255-0)
Supplement: Supplementary file 4 — Additional file 4. Results of ANCOVA analyses. [file 12889_2023_15255_MOESM4_ESM.docx]

**Employees’ preferences on organisational aspects of psychotherapeutic consultation at work by occupational area, company size, requirement levels and supervisor function – a cross-sectional study in Germany**

Fiona Kohl^1^, Peter Angerer^1^, Jeannette Weber^1^

^1^ Institute of Occupational, Social and Environmental Medicine, Centre for Health and Society, Medical Faculty, Heinrich-Heine-University Düsseldorf, Moorenstraße 5, 40225 Düsseldorf, Germany

Corresponding author: Jeannette Weber, Institute of Occupational, Social and Environmental Medicine, Centre for Health and Society, Medical Faculty, Heinrich-Heine-University Düsseldorf, Moorenstraße 5, 40225 Düsseldorf, Germany, Email: Jeannette.Weber@hhu.de

**Additional file 4 – Results of ANCOVA analyses**

**General sample**

Table 1 Results of repeated measures analyses of covariances (RM-ANCOVA) to compare agreement to implementation options regarding type of consultation at work (n = 755)

| **Type of consultation** | | | | | | |
| --- | --- | --- | --- | --- | --- | --- |
| **Effect** | **DFn** | **DFd** | **F** | **p** | **p<.05** | **ges** |
| age | 1 | 720 | 1.472 | 0.225 |  | 0.000994 |
| gender | 2 | 720 | 3.787 | 0.023 | * | 0.005 |
| health insurance | 1 | 720 | 0.748 | 0.387 |  | 0.000505 |
| WHO-5 | 1 | 720 | 0.193 | 0.66 |  | 0.000131 |
| remote work | 1 | 720 | 2.691 | 0.101 |  | 0.002 |
| education | 1 | 720 | 6.135 | 0.013 | * | 0.004 |
| shift work | 1 | 720 | 0.166 | 0.684 |  | 0.000112 |
| implementation option | 2 | 1440 | 767.481 | 1.3E-227 | * | 0.354 |
| age:implementation option | 2 | 1440 | 7.297 | 0.000703 | * | 0.005 |
| gender:implementation option | 4 | 1440 | 2.029 | 0.088 |  | 0.003 |
| health insurance:implementation option | 2 | 1440 | 0.298 | 0.742 |  | 0.000213 |
| WHO-5:implementation option | 2 | 1440 | 0.365 | 0.694 |  | 0.00026 |
| remote work:implementation option | 2 | 1440 | 7.905 | 0.000385 | * | 0.006 |
| education:implementation option | 2 | 1440 | 3.83 | 0.022 | * | 0.003 |
| shift work:implementation option | 2 | 1440 | 0.35 | 0.705 |  | 0.00025 |

*dfd = numerator degrees of freedom in the denominator ; dfn = degrees of freedom in the numerator*; *n = number*

Table 2 Results of repeated measures analyses of covariances (RM-ANCOVA) to compare agreement to implementation options regarding location of consultation at work (n = 755)

| **Location** | | | | | | |
| --- | --- | --- | --- | --- | --- | --- |
| **Effect** | **DFn** | **DFd** | **F** | **p** | **p<.05** | **ges** |
| age | 1 | 720 | 0.176 | 0.675 |  | 0.0000779 |
| gender | 2 | 720 | 0.285 | 0.752 |  | 0.000253 |
| health insurance | 1 | 720 | 1.174 | 0.279 |  | 0.00052 |
| WHO-5 | 1 | 720 | 1.862 | 0.173 |  | 0.000824 |
| remote work | 1 | 720 | 0.003 | 0.957 |  | 0.00000131 |
| education | 1 | 720 | 0.671 | 0.413 |  | 0.000297 |
| shift work | 1 | 720 | 0.049 | 0.825 |  | 0.0000216 |
| implementation option | 1 | 720 | 758.854 | 1.21E-114 | * | 0.418 |
| age:implementation option | 1 | 720 | 0.161 | 0.689 |  | 0.000152 |
| gender:implementation option | 2 | 720 | 1.066 | 0.345 |  | 0.002 |
| health insurance:implementation option | 1 | 720 | 0.138 | 0.711 |  | 0.00013 |
| WHO-5:implementation option | 1 | 720 | 11.739 | 0.000647 | * | 0.011 |
| remote work:implementation option | 1 | 720 | 3.514 | 0.061 |  | 0.003 |
| education:implementation option | 1 | 720 | 0.508 | 0.476 |  | 0.00048 |
| shift work:implementation option | 1 | 720 | 0.192 | 0.661 |  | 0.000182 |

*dfd = numerator degrees of freedom in the denominator ; dfn = degrees of freedom in the numerator*; *n = number*

Table 3 Results of repeated measures analyses of covariances (RM-ANCOVA) to compare agreement to implementation options regarding time of consultation at work (n = 755)

| **Time** | | | | | | |
| --- | --- | --- | --- | --- | --- | --- |
| **Effect** | **DFn** | **DFd** | **F** | **p** | **p<.05** | **ges** |
| age | 1 | 720 | 8,826 | 0,003 | * | 0,003 |
| gender | 2 | 720 | 1,417 | 0,243 |  | 0,000957 |
| health insurance | 1 | 720 | 0,217 | 0,642 |  | 0,0000732 |
| WHO-5 | 1 | 720 | 0,132 | 0,717 |  | 0,0000446 |
| remote work | 1 | 720 | 0,016 | 0,9 |  | 0,00000534 |
| education | 1 | 720 | 3,333 | 0,068 |  | 0,001 |
| shift work | 1 | 720 | 17,104 | 0,0000396 | * | 0,006 |
| Implementation option | 1 | 720 | 6,541 | 0,011 | * | 0,007 |
| age:implementation option | 1 | 720 | 1,455 | 0,228 |  | 0,002 |
| gender:implementation option | 2 | 720 | 0,972 | 0,379 |  | 0,002 |
| health insurance:implementation option | 1 | 720 | 2,263 | 0,133 |  | 0,002 |
| WHO-5:implementation option | 1 | 720 | 0,01 | 0,919 |  | 0,0000108 |
| remote work:implementation option | 1 | 720 | 0,002 | 0,966 |  | 0,00000194 |
| education:implementation option | 1 | 720 | 0,402 | 0,526 |  | 0,000422 |
| shift work:implementation option | 1 | 720 | 0,603 | 0,438 |  | 0,000633 |

*dfd = numerator degrees of freedom in the denominator ; dfn = degrees of freedom in the numerator*; *n = number*

Table 4 Results of repeated measures analyses of covariances (RM-ANCOVA) to compare agreement to implementation options regarding scope of consultation at work (n = 755)

| **Scope** | | | | | | |
| --- | --- | --- | --- | --- | --- | --- |
| **Effect** | **DFn** | **DFd** | **F** | **p** | **p<.05** | **ges** |
| age | 1 | 720 | 0.456 | 0.5 |  | 0.000255 |
| gender | 2 | 720 | 0.651 | 0.522 |  | 0.000728 |
| health insurance | 1 | 720 | 0.259 | 0.611 |  | 0.000145 |
| WHO-5 | 1 | 720 | 1.332 | 0.249 |  | 0.000745 |
| remote work | 1 | 720 | 1.817 | 0.178 |  | 0.001 |
| education | 1 | 720 | 0.976 | 0.323 |  | 0.000547 |
| shift work | 1 | 720 | 1.053 | 0.305 |  | 0.000589 |
| implementation option | 1 | 720 | 1030.049 | 5.36E-141 | * | 0.461 |
| age:implementation option | 1 | 720 | 0.831 | 0.362 |  | 0.000688 |
| gender:implementation option | 2 | 720 | 4.312 | 0.014 | * | 0.007 |
| health insurance:implementation option | 1 | 720 | 0.285 | 0.594 |  | 0.000236 |
| WHO-5:implementation option | 1 | 720 | 2.339 | 0.127 |  | 0.002 |
| remote work:implementation option | 1 | 720 | 3.477 | 0.063 |  | 0.003 |
| education:implementation option | 1 | 720 | 0.692 | 0.406 |  | 0.000574 |
| shift work:implementation option | 1 | 720 | 0.479 | 0.489 |  | 0.000397 |
| shift work:implementation option | 1 | 719 | 0.682 | 0.409 |  | 0.000569 |

*dfd = numerator degrees of freedom in the denominator ; dfn = degrees of freedom in the numerator*; *n = number*

Table 5 Results of repeated measures analyses of covariances (RM-ANCOVA) to compare agreement to implementation options regarding purpose of consultation at work (n = 755)

| **Purpose** | | | | | | |
| --- | --- | --- | --- | --- | --- | --- |
| **Effect** | **DFn** | **DFd** | **F** | **p** | **p<.05** | **ges** |
| age | 1 | 720 | 0.216 | 0.642 |  | 0.000134 |
| gender | 2 | 720 | 3.043 | 0.048 | * | 0.004 |
| health insurance | 1 | 720 | 0.088 | 0.767 |  | 0.0000545 |
| WHO-5 | 1 | 720 | 1.002 | 0.317 |  | 0.000623 |
| remote work | 1 | 720 | 1.303 | 0.254 |  | 0.00081 |
| education | 1 | 720 | 0.184 | 0.668 |  | 0.000115 |
| shift work | 1 | 720 | 1.771 | 0.184 |  | 0.001 |
| implementation option | 3 | 2160 | 45.075 | 3E-28 | * | 0.033 |
| age:implementation option | 3 | 2160 | 5.332 | 0.001 | * | 0.004 |
| gender:implementation option | 6 | 2160 | 1.749 | 0.106 |  | 0.003 |
| health insurance:implementation option | 3 | 2160 | 0.898 | 0.442 |  | 0.000688 |
| WHO-5:implementation option | 3 | 2160 | 3.401 | 0.017 | * | 0.003 |
| remote work:implementation option | 3 | 2160 | 0.729 | 0.535 |  | 0.000558 |
| education:implementation option | 3 | 2160 | 2.827 | 0.037 | * | 0.002 |
| shift work:implementation option | 3 | 2160 | 1.316 | 0.267 |  | 0.001 |

*dfd = numerator degrees of freedom in the denominator ; dfn = degrees of freedom in the numerator*; *n = number*

**Occupational area**

Table 6 Results of repeated measures analyses of covariances (RM-ANCOVA) to compare agreement to implementation options regarding type of consultation at work dependent on occupational area (n = 755)

| **Type of consultation** | | | | | | |
| --- | --- | --- | --- | --- | --- | --- |
| **Effect** | **DFn** | **DFd** | **F** | **p** | **p<.05** | **ges** |
| age | 1 | 713 | 1.192 | 0.275 |  | 0.000815 |
| gender | 2 | 713 | 3.824 | 0.022 | * | 0.005 |
| WHO-5 | 1 | 713 | 0.395 | 0.53 |  | 0.00027 |
| remote work | 1 | 713 | 2.418 | 0.12 |  | 0.002 |
| education | 1 | 713 | 5.122 | 0.024 | * | 0.003 |
| shift work | 1 | 713 | 0.127 | 0.722 |  | 0.0000866 |
| occupational area | 8 | 713 | 0.722 | 0.672 |  | 0.004 |
| implementation option | 2 | 1426 | 27.951 | 1.24E-12 | * | 0.02 |
| age:implementation option | 2 | 1426 | 6.605 | 0.001 | * | 0.005 |
| gender:implementation option | 4 | 1426 | 2.017 | 0.09 |  | 0.003 |
| WHO-5:implementation option | 2 | 1426 | 0.41 | 0.664 |  | 0.000294 |
| remote work:implementation option | 2 | 1426 | 6.447 | 0.002 | * | 0.005 |
| education:implementation option | 2 | 1426 | 3.499 | 0.03 | * | 0.003 |
| shift work:implementation option | 2 | 1426 | 0.245 | 0.782 |  | 0.000176 |
| occupational area:implementation option | 16 | 1426 | 1.108 | 0.342 |  | 0.006 |

*dfd = numerator degrees of freedom in the denominator ; dfn = degrees of freedom in the numerator*; *n = number*

Table 7 Results of repeated measures analyses of covariances (RM-ANCOVA) to compare agreement to implementation options regarding location of consultation at work dependent on occupational area (n = 755)

| **Location** | | | | | | |
| --- | --- | --- | --- | --- | --- | --- |
| **Effect** | **DFn** | **DFd** | **F** | **p** | **p<.05** | **ges** |
| age | 1 | 713 | 0.308 | 0.579 |  | 0.000139 |
| gender | 2 | 713 | 0.2 | 0.819 |  | 0.000181 |
| WHO-5 | 1 | 713 | 1.892 | 0.169 |  | 0.000856 |
| remote work | 1 | 713 | 0.106 | 0.745 |  | 0.0000479 |
| education | 1 | 713 | 0.513 | 0.474 |  | 0.000232 |
| shift work | 1 | 713 | 0.184 | 0.668 |  | 0.0000833 |
| occupational area | 8 | 713 | 0.54 | 0.827 |  | 0.002 |
| implementation option | 1 | 713 | 23.92 | 0.00000124 | * | 0.022 |
| age:implementation option | 1 | 713 | 0.672 | 0.413 |  | 0.000637 |
| gender:implementation option | 2 | 713 | 0.983 | 0.375 |  | 0.002 |
| WHO-5:implementation option | 1 | 713 | 9.61 | 0.002 | * | 0.009 |
| remote work:implementation option | 1 | 713 | 1.665 | 0.197 |  | 0.002 |
| education:implementation option | 1 | 713 | 0.773 | 0.38 |  | 0.000733 |
| shift work:implementation option | 1 | 713 | 0.258 | 0.611 |  | 0.000245 |
| occupational area:implementation option | 8 | 713 | 2.197 | 0.026 | * | 0.016 |

*dfd = numerator degrees of freedom in the denominator ; dfn = degrees of freedom in the numerator*; *n = number*

Table 8 Results of repeated measures analyses of covariances (RM-ANCOVA) to compare agreement to implementation options regarding time of consultation at work dependent on occupational area (n = 755)

| **Time** | | | | | | |
| --- | --- | --- | --- | --- | --- | --- |
| **Effect** | **DFn** | **DFd** | **F** | **p** | **p<.05** | **ges** |
| age | 1 | 713 | 8,387 | 0,004 | * | 0,003 |
| gender | 2 | 713 | 0,975 | 0,378 |  | 0,000666 |
| WHO-5 | 1 | 713 | 0,264 | 0,608 |  | 0,0000901 |
| remote work | 1 | 713 | 0,028 | 0,867 |  | 0,00000953 |
| education | 1 | 713 | 3,067 | 0,08 |  | 0,001 |
| shift work | 1 | 713 | 14,636 | 0,000142 | * | 0,005 |
| occupational area | 8 | 713 | 0,877 | 0,535 |  | 0,002 |
| implementation option | 1 | 713 | 0,238 | 0,626 |  | 0,000252 |
| age:implementation option | 1 | 713 | 1,653 | 0,199 |  | 0,002 |
| gender:implementation option | 2 | 713 | 1,023 | 0,36 |  | 0,002 |
| WHO-5:implementation option | 1 | 713 | 0,104 | 0,747 |  | 0,00011 |
| remote work:implementation option | 1 | 713 | 0,000637 | 0,98 |  | 6,76E-07 |
| education:implementation option | 1 | 713 | 0,051 | 0,822 |  | 0,0000536 |
| shift work:implementation option | 1 | 713 | 0,325 | 0,569 |  | 0,000345 |
| occupational area:implementation option | 8 | 713 | 1,246 | 0,269 |  | 0,01 |

*dfd = numerator degrees of freedom in the denominator ; dfn = degrees of freedom in the numerator*; *n = number*

Table 9 Results of repeated measures analyses of covariances (RM-ANCOVA) to compare agreement to implementation options regarding scope of consultation at work dependent on occupational area (n = 755)

| **Scope** | | | | | | |
| --- | --- | --- | --- | --- | --- | --- |
| **Effect** | **DFn** | **DFd** | **F** | **p** | **p<.05** | **ges** |
| age | 1 | 713 | 0.308 | 0.579 |  | 0.000139 |
| gender | 2 | 713 | 0.2 | 0.819 |  | 0.000181 |
| WHO-5 | 1 | 713 | 1.892 | 0.169 |  | 0.000856 |
| remote work | 1 | 713 | 0.106 | 0.745 |  | 0.0000479 |
| education | 1 | 713 | 0.513 | 0.474 |  | 0.000232 |
| shift work | 1 | 713 | 0.184 | 0.668 |  | 0.0000833 |
| occupational area | 8 | 713 | 0.54 | 0.827 |  | 0.002 |
| implementation option | 1 | 713 | 23.92 | 0.00000124 | * | 0.022 |
| age:implementation option | 1 | 713 | 0.672 | 0.413 |  | 0.000637 |
| gender:implementation option | 2 | 713 | 0.983 | 0.375 |  | 0.002 |
| WHO-5:implementation option | 1 | 713 | 9.61 | 0.002 | * | 0.009 |
| remote work:implementation option | 1 | 713 | 1.665 | 0.197 |  | 0.002 |
| education:implementation option | 1 | 713 | 0.773 | 0.38 |  | 0.000733 |
| shift work:implementation option | 1 | 713 | 0.258 | 0.611 |  | 0.000245 |
| occupational area:implementation option | 8 | 713 | 2.197 | 0.026 | * | 0.016 |

*dfd = numerator degrees of freedom in the denominator ; dfn = degrees of freedom in the numerator*; *n = number*

Table 10 Results of repeated measures analyses of covariances (RM-ANCOVA) to compare agreement to implementation options regarding purpose of consultation at work dependent on occupational area (n = 755)

| **Purpose** | | | | | | |
| --- | --- | --- | --- | --- | --- | --- |
| **Effect** | **DFn** | **DFd** | **F** | **p** | **p<.05** | **ges** |
| age | 1 | 713 | 0.117 | 0.733 |  | 0.0000735 |
| gender | 2 | 713 | 2.974 | 0.052 |  | 0.004 |
| WHO-5 | 1 | 713 | 1.056 | 0.304 |  | 0.000664 |
| remote work | 1 | 713 | 1.027 | 0.311 |  | 0.000646 |
| education | 1 | 713 | 0.389 | 0.533 |  | 0.000245 |
| shift work | 1 | 713 | 1.647 | 0.2 |  | 0.001 |
| occupational area | 8 | 713 | 0.756 | 0.642 |  | 0.004 |
| implementation option | 3 | 2139 | 2.938 | 0.032 | * | 0.002 |
| age:implementation option | 3 | 2139 | 5.1 | 0.002 | * | 0.004 |
| gender:implementation option | 6 | 2139 | 1.864 | 0.083 |  | 0.003 |
| WHO-5:implementation option | 3 | 2139 | 3.009 | 0.029 | * | 0.002 |
| remote work:implementation option | 3 | 2139 | 0.671 | 0.57 |  | 0.000519 |
| education:implementation option | 3 | 2139 | 2.841 | 0.037 | * | 0.002 |
| shift work:implementation option | 3 | 2139 | 1.531 | 0.204 |  | 0.001 |
| occupational area:implementation option | 24 | 2139 | 1.05 | 0.396 |  | 0.006 |

*dfd = numerator degrees of freedom in the denominator ; dfn = degrees of freedom in the numerator*; *n = number*

**Company size**

Table 11 Results of repeated measures analyses of covariances (RM-ANCOVA) to compare agreement to implementation options regarding type of consultation at work dependent on company size (n = 755)

| **Type of consultation** | | | | | | |
| --- | --- | --- | --- | --- | --- | --- |
| **Effect** | **DFn** | **DFd** | **F** | **p** | **p<.05** | **ges** |
| age | 1 | 717 | 1.409 | 0.236 |  | 0.000957 |
| gender | 2 | 717 | 3.726 | 0.025 | * | 0.005 |
| WHO-5 | 1 | 717 | 0.197 | 0.657 |  | 0.000134 |
| remote work | 1 | 717 | 2.356 | 0.125 |  | 0.002 |
| education | 1 | 717 | 6.178 | 0.013 | * | 0.004 |
| shift work | 1 | 717 | 0.04 | 0.842 |  | 0.000027 |
| company size | 4 | 717 | 0.339 | 0.851 |  | 0.000922 |
| implementation option | 2 | 1434 | 28.236 | 9.39E-13 | * | 0.02 |
| age:implementation option | 2 | 1434 | 6.796 | 0.001 | * | 0.005 |
| gender:implementation option | 4 | 1434 | 1.776 | 0.131 |  | 0.003 |
| WHO-5:implementation option | 2 | 1434 | 0.364 | 0.695 |  | 0.00026 |
| remote work:implementation option | 2 | 1434 | 7.637 | 0.000502 | * | 0.005 |
| education:implementation option | 2 | 1434 | 3.888 | 0.021 | * | 0.003 |
| shift work:implementation option | 2 | 1434 | 0.311 | 0.733 |  | 0.000222 |
| company size:implementation option | 8 | 1434 | 0.721 | 0.673 |  | 0.002 |

*dfd = numerator degrees of freedom in the denominator ; dfn = degrees of freedom in the numerator*; *n = number*

Table 12 Results of repeated measures analyses of covariances (RM-ANCOVA) to compare agreement to implementation options regarding location of consultation at work dependent on company size (n = 755)

| **Location** | | | | | | |
| --- | --- | --- | --- | --- | --- | --- |
| **Effect** | **DFn** | **DFd** | **F** | **p** | **p<.05** | **ges** |
| age | 1 | 717 | 0.38 | 0.538 |  | 0.000169 |
| gender | 2 | 717 | 0.36 | 0.698 |  | 0.00032 |
| WHO-5 | 1 | 717 | 2.403 | 0.122 |  | 0.001 |
| remote work | 1 | 717 | 0.244 | 0.621 |  | 0.000109 |
| education | 1 | 717 | 1.164 | 0.281 |  | 0.000517 |
| shift work | 1 | 717 | 0.03 | 0.862 |  | 0.0000134 |
| company size | 4 | 717 | 3.403 | 0.009 | * | 0.006 |
| implementation option | 1 | 717 | 22.463 | 0.00000258 | * | 0.021 |
| age:implementation option | 1 | 717 | 0.385 | 0.535 |  | 0.000365 |
| gender:implementation option | 2 | 717 | 1.587 | 0.205 |  | 0.003 |
| WHO-5:implementation option | 1 | 717 | 12.969 | 0.000339 | * | 0.012 |
| remote work:implementation option | 1 | 717 | 4.814 | 0.029 | * | 0.005 |
| education:implementation option | 1 | 717 | 0.223 | 0.637 |  | 0.000212 |
| shift work:implementation option | 1 | 717 | 0.746 | 0.388 |  | 0.000708 |
| company size:implementation option | 4 | 717 | 3.19 | 0.013 | * | 0.012 |

*dfd = numerator degrees of freedom in the denominator ; dfn = degrees of freedom in the numerator*; *n = number*

Table 13 Results of repeated measures analyses of covariances (RM-ANCOVA) to compare agreement to implementation options regarding time of consultation at work dependent on company size (n = 755)

| **Time** | | | | | | |
| --- | --- | --- | --- | --- | --- | --- |
| **Effect** | **DFn** | **DFd** | **F** | **p** | **p<.05** | **ges** |
| age | 1 | 717 | 9,039 | 0,003 | * | 0,003 |
| gender | 2 | 717 | 1,473 | 0,23 |  | 0,000993 |
| WHO-5 | 1 | 717 | 0,073 | 0,787 |  | 0,0000248 |
| remote work | 1 | 717 | 0,014 | 0,906 |  | 0,00000474 |
| education | 1 | 717 | 3,384 | 0,066 |  | 0,001 |
| shift work | 1 | 717 | 19,96 | 0,00000919 | * | 0,007 |
| company size | 4 | 717 | 2,024 | 0,089 |  | 0,003 |
| implementation option | 1 | 717 | 0,172 | 0,678 |  | 0,000182 |
| age:implementation option | 1 | 717 | 1,368 | 0,243 |  | 0,001 |
| gender:implementation option | 2 | 717 | 1,084 | 0,339 |  | 0,002 |
| WHO-5:implementation option | 1 | 717 | 0,034 | 0,853 |  | 0,0000361 |
| remote work:implementation option | 1 | 717 | 0,002 | 0,963 |  | 0,00000222 |
| education:implementation option | 1 | 717 | 0,363 | 0,547 |  | 0,000384 |
| shift work:implementation option | 1 | 717 | 0,515 | 0,473 |  | 0,000544 |
| company size:implementation option | 4 | 717 | 1,098 | 0,356 |  | 0,005 |

*dfd = numerator degrees of freedom in the denominator ; dfn = degrees of freedom in the numerator*; *n = number*

Table 14 Results of repeated measures analyses of covariances (RM-ANCOVA) to compare agreement to implementation options regarding scope of consultation at work dependent on company size (n = 755)

| **Scope** | | | | | | |
| --- | --- | --- | --- | --- | --- | --- |
| **Effect** | **DFn** | **DFd** | **F** | **p** | **p<.05** | **ges** |
| age | 1 | 717 | 0.577 | 0.448 |  | 0.000323 |
| gender | 2 | 717 | 0.331 | 0.718 |  | 0.00037 |
| WHO-5 | 1 | 717 | 1.169 | 0.28 |  | 0.000654 |
| remote work | 1 | 717 | 0.943 | 0.332 |  | 0.000528 |
| education | 1 | 717 | 1.163 | 0.281 |  | 0.00065 |
| shift work | 1 | 717 | 1.603 | 0.206 |  | 0.000896 |
| company size | 4 | 717 | 1.747 | 0.138 |  | 0.004 |
| implementation option | 1 | 717 | 17.712 | 0.000029 | * | 0.015 |
| age:implementation option | 1 | 717 | 0.751 | 0.386 |  | 0.000627 |
| gender:implementation option | 2 | 717 | 4.235 | 0.015 | * | 0.007 |
| WHO-5:implementation option | 1 | 717 | 2.247 | 0.134 |  | 0.002 |
| remote work:implementation option | 1 | 717 | 3.175 | 0.075 |  | 0.003 |
| education:implementation option | 1 | 717 | 0.708 | 0.4 |  | 0.000591 |
| shift work:implementation option | 1 | 717 | 0.414 | 0.52 |  | 0.000346 |
| company size:implementation option | 4 | 717 | 0.216 | 0.93 |  | 0.000721 |

*dfd = numerator degrees of freedom in the denominator ; dfn = degrees of freedom in the numerator*; *n = number*

Table 15 Results of repeated measures analyses of covariances (RM-ANCOVA) to compare agreement to implementation options regarding purpose of consultation at work dependent on company size (n = 755)

| **Purpose** | | | | | | |
| --- | --- | --- | --- | --- | --- | --- |
| **Effect** | **DFn** | **DFd** | **F** | **p** | **p<.05** | **ges** |
| age | 1 | 717 | 0.364 | 0.547 |  | 0.000228 |
| gender | 2 | 717 | 3.377 | 0.035 | * | 0.004 |
| WHO-5 | 1 | 717 | 0.841 | 0.359 |  | 0.000528 |
| remote work | 1 | 717 | 1.948 | 0.163 |  | 0.001 |
| education | 1 | 717 | 0.363 | 0.547 |  | 0.000228 |
| shift work | 1 | 717 | 2.813 | 0.094 |  | 0.002 |
| company size | 4 | 717 | 1.512 | 0.197 |  | 0.004 |
| implementation option | 3 | 2151 | 3.064 | 0.027 | * | 0.002 |
| age:implementation option | 3 | 2151 | 5.372 | 0.001 | * | 0.004 |
| gender:implementation option | 6 | 2151 | 2.23 | 0.038 | * | 0.003 |
| WHO-5:implementation option | 3 | 2151 | 3.536 | 0.014 | * | 0.003 |
| remote work:implementation option | 3 | 2151 | 0.806 | 0.491 |  | 0.000618 |
| education:implementation option | 3 | 2151 | 2.953 | 0.031 | * | 0.002 |
| shift work:implementation option | 3 | 2151 | 0.877 | 0.452 |  | 0.000672 |
| company size:implementation option | 12 | 2151 | 3.176 | 0.000162 | * | 0.01 |

*dfd = numerator degrees of freedom in the denominator ; dfn = degrees of freedom in the numerator*; *n = number*

**Supervisor function**

Table 16 Results of repeated measures analyses of covariances (RM-ANCOVA) to compare agreement to implementation options regarding type of consultation at work dependent on supervisor function (n = 755)

| **Type of consultation** | | | | | | |
| --- | --- | --- | --- | --- | --- | --- |
| **Effect** | **DFn** | **DFd** | **F** | **p** | **p<.05** | **ges** |
| age | 1 | 720 | 1.339 | 0.248 |  | 0.000905 |
| gender | 2 | 720 | 3.68 | 0.026 | * | 0.005 |
| WHO-5 | 1 | 720 | 0.199 | 0.656 |  | 0.000134 |
| remote work | 1 | 720 | 2.85 | 0.092 |  | 0.002 |
| education | 1 | 720 | 6.437 | 0.011 | * | 0.004 |
| shift work | 1 | 720 | 0.144 | 0.704 |  | 0.0000976 |
| supervisor function | 1 | 720 | 0.005 | 0.946 |  | 0.00000309 |
| implementation option | 2 | 1440 | 28.985 | 4.56E-13 | * | 0.02 |
| age:implementation option | 2 | 1440 | 7.169 | 0.000798 | * | 0.005 |
| gender:implementation option | 4 | 1440 | 2.064 | 0.083 |  | 0.003 |
| WHO-5:implementation option | 2 | 1440 | 0.338 | 0.713 |  | 0.000241 |
| remote work:implementation option | 2 | 1440 | 8.118 | 0.000312 | * | 0.006 |
| education:implementation option | 2 | 1440 | 4.111 | 0.017 | * | 0.003 |
| shift work:implementation option | 2 | 1440 | 0.366 | 0.693 |  | 0.000261 |
| supervisor function:implementation option | 2 | 1440 | 0.486 | 0.615 |  | 0.000346 |

*dfd = numerator degrees of freedom in the denominator ; dfn = degrees of freedom in the numerator*; *n = number*

Table 17 Results of repeated measures analyses of covariances (RM-ANCOVA) to compare agreement to implementation options regarding location of consultation at work dependent on supervisor function (n = 755)

| **Location** | | | | | | |
| --- | --- | --- | --- | --- | --- | --- |
| **Effect** | **DFn** | **DFd** | **F** | **p** | **p<.05** | **ges** |
| age | 1 | 720 | 0.074 | 0.786 |  | 0.0000327 |
| gender | 2 | 720 | 0.258 | 0.772 |  | 0.000229 |
| WHO-5 | 1 | 720 | 2.153 | 0.143 |  | 0.000953 |
| remote work | 1 | 720 | 0.026 | 0.872 |  | 0.0000116 |
| education | 1 | 720 | 1.007 | 0.316 |  | 0.000446 |
| shift work | 1 | 720 | 0.014 | 0.904 |  | 0.00000638 |
| supervisor function | 1 | 720 | 4.604 | 0.032 | * | 0.002 |
| implementation option | 1 | 720 | 26.571 | 3.28E-07 | * | 0.025 |
| age:implementation option | 1 | 720 | 0.052 | 0.819 |  | 0.0000495 |
| gender:implementation option | 2 | 720 | 0.855 | 0.426 |  | 0.002 |
| WHO-5:implementation option | 1 | 720 | 12.388 | 0.000459 | * | 0.012 |
| remote work:implementation option | 1 | 720 | 3.788 | 0.052 |  | 0.004 |
| education:implementation option | 1 | 720 | 0.35 | 0.554 |  | 0.000331 |
| shift work:implementation option | 1 | 720 | 0.303 | 0.582 |  | 0.000286 |
| supervisor function:implementation option | 1 | 720 | 4.025 | 0.045 | * | 0.004 |

*dfd = numerator degrees of freedom in the denominator ; dfn = degrees of freedom in the numerator*; *n = number*

Table 18 Results of repeated measures analyses of covariances (RM-ANCOVA) to compare agreement to implementation options regarding time of consultation at work dependent on supervisor function (n = 755)

| **Time** | | | | | | |
| --- | --- | --- | --- | --- | --- | --- |
| **Effect** | **DFn** | **DFd** | **F** | **p** | **p<.05** | **ges** |
| age | 1 | 720 | 8,36 | 0,004 | * | 0,003 |
| gender | 2 | 720 | 1,492 | 0,226 |  | 0,001 |
| WHO-5 | 1 | 720 | 0,143 | 0,706 |  | 0,0000481 |
| remote work | 1 | 720 | 0,02 | 0,888 |  | 0,00000675 |
| education | 1 | 720 | 3,306 | 0,069 |  | 0,001 |
| shift work | 1 | 720 | 17,396 | 0,0000341 | * | 0,006 |
| supervisor function | 1 | 720 | 0,229 | 0,632 |  | 0,0000775 |
| implementation option | 1 | 720 | 0,126 | 0,723 |  | 0,000132 |
| age:implementation option | 1 | 720 | 1,336 | 0,248 |  | 0,001 |
| gender:implementation option | 2 | 720 | 0,958 | 0,384 |  | 0,002 |
| WHO-5:implementation option | 1 | 720 | 0,000926 | 0,976 |  | 9,73E-07 |
| remote work:implementation option | 1 | 720 | 0,005 | 0,945 |  | 0,00000501 |
| education:implementation option | 1 | 720 | 0,216 | 0,643 |  | 0,000226 |
| shift work:implementation option | 1 | 720 | 0,663 | 0,416 |  | 0,000697 |
| supervisor function:implementation option | 1 | 720 | 1,714 | 0,191 |  | 0,002 |

*dfd = numerator degrees of freedom in the denominator ; dfn = degrees of freedom in the numerator*; *n = number*

Table 19 Results of repeated measures analyses of covariances (RM-ANCOVA) to compare agreement to implementation options regarding scope of consultation at work dependent on supervisor function (n = 755)

| **Scope** | | | | | | |
| --- | --- | --- | --- | --- | --- | --- |
| **Effect** | **DFn** | **DFd** | **F** | **p** | **p<.05** | **ges** |
| age | 1 | 720 | 0.577 | 0.448 |  | 0.000323 |
| gender | 2 | 720 | 0.557 | 0.573 |  | 0.000625 |
| WHO-5 | 1 | 720 | 1.377 | 0.241 |  | 0.000771 |
| remote work | 1 | 720 | 1.782 | 0.182 |  | 0.000999 |
| education | 1 | 720 | 0.991 | 0.32 |  | 0.000555 |
| shift work | 1 | 720 | 0.948 | 0.331 |  | 0.000531 |
| supervisor function | 1 | 720 | 0.39 | 0.532 |  | 0.000219 |
| implementation option | 1 | 720 | 16.266 | 0.0000609 | * | 0.013 |
| age:implementation option | 1 | 720 | 1.161 | 0.282 |  | 0.000961 |
| gender:implementation option | 2 | 720 | 3.891 | 0.021 | * | 0.006 |
| WHO-5:implementation option | 1 | 720 | 2.178 | 0.14 |  | 0.002 |
| remote work:implementation option | 1 | 720 | 3.503 | 0.062 |  | 0.003 |
| education:implementation option | 1 | 720 | 0.752 | 0.386 |  | 0.000623 |
| shift work:implementation option | 1 | 720 | 0.348 | 0.556 |  | 0.000288 |
| supervisor function:implementation option | 1 | 720 | 2.149 | 0.143 |  | 0.002 |

*dfd = numerator degrees of freedom in the denominator ; dfn = degrees of freedom in the numerator*; *n = number*

Table 20 Results of repeated measures analyses of covariances (RM-ANCOVA) to compare agreement to implementation options regarding purpose of consultation at work dependent on supervisor function (n = 755)

| **Purpose** | | | | | | |
| --- | --- | --- | --- | --- | --- | --- |
| **Effect** | **DFn** | **DFd** | **F** | **p** | **p<.05** | **ges** |
| age | 1 | 720 | 0.104 | 0.748 |  | 0.0000643 |
| gender | 2 | 720 | 2.968 | 0.052 |  | 0.004 |
| WHO-5 | 1 | 720 | 0.866 | 0.352 |  | 0.000537 |
| remote work | 1 | 720 | 1.434 | 0.231 |  | 0.000889 |
| education | 1 | 720 | 0.283 | 0.595 |  | 0.000175 |
| shift work | 1 | 720 | 2.026 | 0.155 |  | 0.001 |
| supervisor function | 1 | 720 | 2.732 | 0.099 |  | 0.002 |
| implementation option | 3 | 2160 | 2.655 | 0.047 | * | 0.002 |
| age:implementation option | 3 | 2160 | 5.122 | 0.002 | * | 0.004 |
| gender:implementation option | 6 | 2160 | 1.797 | 0.096 |  | 0.003 |
| WHO-5:implementation option | 3 | 2160 | 3.361 | 0.018 | * | 0.003 |
| remote work:implementation option | 3 | 2160 | 0.653 | 0.581 |  | 0.000502 |
| education:implementation option | 3 | 2160 | 2.913 | 0.033 | * | 0.002 |
| shift work:implementation option | 3 | 2160 | 1.384 | 0.246 |  | 0.001 |
| supervisor function:implementation option | 3 | 2160 | 0.343 | 0.794 |  | 0.000263 |

*dfd = numerator degrees of freedom in the denominator ; dfn = degrees of freedom in the numerator*; *n = number*

**Requirement level**

Table 21 Results of repeated measures analyses of covariances (RM-ANCOVA) to compare agreement to implementation options regarding type of consultation at work dependent on requirement level (n = 755)

| **Type of consultation** | | | | | | |
| --- | --- | --- | --- | --- | --- | --- |
| **Effect** | **DFn** | **DFd** | **F** | **p** | **p<.05** | **ges** |
| age | 1 | 718 | 2.246 | 0.134 |  | 0.002 |
| gender | 2 | 718 | 3.656 | 0.026 | * | 0.005 |
| WHO-5 | 1 | 718 | 0.335 | 0.563 |  | 0.000227 |
| remote work | 1 | 718 | 2.212 | 0.137 |  | 0.001 |
| education | 1 | 718 | 1.988 | 0.159 |  | 0.001 |
| shift work | 1 | 718 | 0.249 | 0.618 |  | 0.000169 |
| requirement level | 3 | 718 | 0.923 | 0.429 |  | 0.002 |
| implementation option | 2 | 1436 | 22.219 | 3.14E-10 | * | 0.016 |
| age:implementation option | 2 | 1436 | 7.156 | 0.000809 | * | 0.005 |
| gender:implementation option | 4 | 1436 | 1.974 | 0.096 |  | 0.003 |
| WHO-5:implementation option | 2 | 1436 | 0.288 | 0.75 |  | 0.000205 |
| remote work:implementation option | 2 | 1436 | 7.235 | 0.000747 | * | 0.005 |
| education:implementation option | 2 | 1436 | 1.391 | 0.249 |  | 0.000993 |
| shift work:implementation option | 2 | 1436 | 0.365 | 0.694 |  | 0.000261 |
| requirement level:implementation option | 6 | 1436 | 1.159 | 0.326 |  | 0.002 |

*dfd = numerator degrees of freedom in the denominator ; dfn = degrees of freedom in the numerator*; *n = number*

Table 22 Results of repeated measures analyses of covariances (RM-ANCOVA) to compare agreement to implementation options regarding location of consultation at work dependent on requirement level (n = 755)

| **Location** | | | | | | |
| --- | --- | --- | --- | --- | --- | --- |
| **Effect** | **DFn** | **DFd** | **F** | **p** | **p<.05** | **ges** |
| age | 1 | 718 | 0.074 | 0.785 |  | 0.000033 |
| gender | 2 | 718 | 0.18 | 0.835 |  | 0.00016 |
| WHO-5 | 1 | 718 | 2.309 | 0.129 |  | 0.001 |
| remote work | 1 | 718 | 0.002 | 0.968 |  | 7.04E-07 |
| education | 1 | 718 | 0.397 | 0.529 |  | 0.000176 |
| shift work | 1 | 718 | 0.03 | 0.862 |  | 0.0000134 |
| requirement level | 3 | 718 | 0.868 | 0.457 |  | 0.001 |
| implementation option | 1 | 718 | 24.308 | 0.00000102 | * | 0.023 |
| age:implementation option | 1 | 718 | 0.06 | 0.807 |  | 0.0000566 |
| gender:implementation option | 2 | 718 | 0.919 | 0.399 |  | 0.002 |
| WHO-5:implementation option | 1 | 718 | 11.963 | 0.000575 | * | 0.011 |
| remote work:implementation option | 1 | 718 | 3.108 | 0.078 |  | 0.003 |
| education:implementation option | 1 | 718 | 1.257 | 0.263 |  | 0.001 |
| shift work:implementation option | 1 | 718 | 0.272 | 0.602 |  | 0.000258 |
| requirement level:implementation option | 3 | 718 | 0.341 | 0.796 |  | 0.000969 |

*dfd = numerator degrees of freedom in the denominator ; dfn = degrees of freedom in the numerator*; *n = number*

Table 23 Results of repeated measures analyses of covariances (RM-ANCOVA) to compare agreement to implementation options regarding time of consultation at work dependent on requirement level (n = 755)

| **Time** | | | | | | |
| --- | --- | --- | --- | --- | --- | --- |
| **Effect** | **DFn** | **DFd** | **F** | **p** | **p<.05** | **ges** |
| age | 1 | 718 | 11,755 | 0,000641 | * | 0,004 |
| gender | 2 | 718 | 1,566 | 0,21 |  | 0,001 |
| WHO-5 | 1 | 718 | 0,012 | 0,914 |  | 0,00000394 |
| remote work | 1 | 718 | 0,021 | 0,886 |  | 0,00000695 |
| education | 1 | 718 | 6,81 | 0,009 | * | 0,002 |
| shift work | 1 | 718 | 15,347 | 0,000098 | * | 0,005 |
| requirement level | 3 | 718 | 2,097 | 0,099 |  | 0,002 |
| implementation option | 1 | 718 | 3,14E-07 | 1 |  | 3,32E-10 |
| age:implementation option | 1 | 718 | 1,283 | 0,258 |  | 0,001 |
| gender:implementation option | 2 | 718 | 0,87 | 0,419 |  | 0,002 |
| WHO-5:implementation option | 1 | 718 | 0,000693 | 0,979 |  | 7,32E-07 |
| remote work:implementation option | 1 | 718 | 0,007 | 0,932 |  | 0,00000767 |
| education:implementation option | 1 | 718 | 1,131 | 0,288 |  | 0,001 |
| shift work:implementation option | 1 | 718 | 0,772 | 0,38 |  | 0,000814 |
| requirement level:implementation option | 3 | 718 | 0,897 | 0,443 |  | 0,003 |

*dfd = numerator degrees of freedom in the denominator ; dfn = degrees of freedom in the numerator*; *n = number*

Table 24 Results of repeated measures analyses of covariances (RM-ANCOVA) to compare agreement to implementation options regarding scope of consultation at work dependent on requirement level (n = 755)

| **Scope** | | | | | | |
| --- | --- | --- | --- | --- | --- | --- |
| **Effect** | **DFn** | **DFd** | **F** | **p** | **p<.05** | **ges** |
| age | 1 | 718 | 1.32 | 0.251 |  | 0.000739 |
| gender | 2 | 718 | 0.47 | 0.625 |  | 0.000526 |
| WHO-5 | 1 | 718 | 1.718 | 0.19 |  | 0.000961 |
| remote work | 1 | 718 | 1.161 | 0.282 |  | 0.00065 |
| education | 1 | 718 | 3.069 | 0.08 |  | 0.002 |
| shift work | 1 | 718 | 0.769 | 0.381 |  | 0.00043 |
| requirement level | 3 | 718 | 1.624 | 0.182 |  | 0.003 |
| implementation option | 1 | 718 | 13.192 | 0.000301 | * | 0.011 |
| age:implementation option | 1 | 718 | 1.124 | 0.289 |  | 0.000935 |
| gender:implementation option | 2 | 718 | 4.204 | 0.015 | * | 0.007 |
| WHO-5:implementation option | 1 | 718 | 2.211 | 0.137 |  | 0.002 |
| remote work:implementation option | 1 | 718 | 3.132 | 0.077 |  | 0.003 |
| education:implementation option | 1 | 718 | 0.264 | 0.607 |  | 0.00022 |
| shift work:implementation option | 1 | 718 | 0.457 | 0.499 |  | 0.000381 |
| requirement level:implementation option | 3 | 718 | 0.397 | 0.755 |  | 0.000991 |

*dfd = numerator degrees of freedom in the denominator ; dfn = degrees of freedom in the numerator*; *n = number*

Table 25 Results of repeated measures analyses of covariances (RM-ANCOVA) to compare agreement to implementation options regarding purpose of consultation at work dependent on requirement level (n = 755)

| **Purpose** | | | | | | |
| --- | --- | --- | --- | --- | --- | --- |
| **Effect** | **DFn** | **DFd** | **F** | **p** | **p<.05** | **ges** |
| age | 1 | 718 | 0.313 | 0.576 |  | 0.000195 |
| gender | 2 | 718 | 2.797 | 0.062 |  | 0.003 |
| WHO-5 | 1 | 718 | 1.308 | 0.253 |  | 0.000814 |
| remote work | 1 | 718 | 1.442 | 0.23 |  | 0.000897 |
| education | 1 | 718 | 0.19 | 0.663 |  | 0.000118 |
| shift work | 1 | 718 | 1.462 | 0.227 |  | 0.000909 |
| requirement level | 3 | 718 | 1.889 | 0.13 |  | 0.004 |
| implementation option | 3 | 2154 | 2.366 | 0.069 |  | 0.002 |
| age:implementation option | 3 | 2154 | 5.114 | 0.002 | * | 0.004 |
| gender:implementation option | 6 | 2154 | 1.596 | 0.144 |  | 0.002 |
| WHO-5:implementation option | 3 | 2154 | 3.162 | 0.024 | * | 0.002 |
| remote work:implementation option | 3 | 2154 | 0.447 | 0.72 |  | 0.000344 |
| education:implementation option | 3 | 2154 | 2.6 | 0.051 |  | 0.002 |
| shift work:implementation option | 3 | 2154 | 1.468 | 0.221 |  | 0.001 |
| requirement level:implementation option | 9 | 2154 | 1.276 | 0.245 |  | 0.003 |

*dfd = numerator degrees of freedom in the denominator ; dfn = degrees of freedom in the numerator*; *n = number*
