# Supplementary material for: Employees’ preferences on organisational aspects of psychotherapeutic consultation at work by occupational area, company size, requirement levels and supervisor function – a cross-sectional study in Germany
Source: BMC Public Health. 2023 Feb 16;23:347. doi: 10.1186/s12889-023-15255-0 (PMC9932407; doi:10.1186/s12889-023-15255-0)
Supplement: Supplementary file 5 — Additional file 5. Results of sensitivity analyses. [file 12889_2023_15255_MOESM5_ESM.docx]

**Employees’ preferences on organisational aspects of psychotherapeutic consultation at work by occupational area, company size, requirement levels and supervisor function – a cross-sectional study in Germany**

Fiona Kohl^1^, Peter Angerer^1^, Jeannette Weber^1^

^1^ Institute of Occupational, Social and Environmental Medicine, Centre for Health and Society, Medical Faculty, Heinrich-Heine-University Düsseldorf, Moorenstraße 5, 40225 Düsseldorf, Germany

Corresponding author: Jeannette Weber, Institute of Occupational, Social and Environmental Medicine, Centre for Health and Society, Medical Faculty, Heinrich-Heine-University Düsseldorf, Moorenstraße 5, 40225 Düsseldorf, Germany, Email: Jeannette.Weber@hhu.de

**Additional file 5 – Results of sensitivity analyses**

Table 1 Results of repeated measures analyses of variance (RM-ANOVA) for comparison between different implementation options of psychotherapeutic consultation at work in a subgroup of employees with depressive symptoms^1^ (n = 510)

|  |  | **Effect of implementation options (within variable) on preference choices (repeated measures ANOVA)** | |
| --- | --- | --- | --- |
|  | **mean (SD)** | **F (dfn, dfd)** | **p-value** |
| **Type of consultation** |  |  |  |
| In-person | 4.80 (0.58) | F (2, 1018) = 548.131 | **<0.001** |
| Telephone | 3.03 (1.22) |  |  |
| Video-based | 3.30 (1.24) |  |  |
| **Location** |  |  |  |
| Outside company premises | 4.51 (0.82) | F (1, 509) = 670.737 | **< 0.001** |
| On company premises | 2.53 (1.26) |  |  |
| **Time** |  |  |  |
| Outside working hours | 3.61 (1.92) | F (1, 509) = 4.086 | **0.044** |
| Within working hours | 3.41 (1.32) |  |  |
| **Scope** |  |  |  |
| Diagnostic only | 2.81 (1.09) | F (1, 509) = 828.128 | **<0.001** |
| Diagnostic + treatment | 4.58 (0.71) |  |  |
| **Purpose** |  |  |  |
| Occupational burden | 4.45 (0.94) | F (1, 1527) = 26.029 | **0.028** |
| Maintain work ability | 4.48 (0.89) |  |  |
| Private burden | 4.05 (1.06) |  |  |
| Occupational reintegration | 4.32 (1.09) |  |  |
| **Accepted distance** | n (%) |  |  |
| < 15 Min | 65 (13) | n.a. |  |
| 15 – 30 Min | 312 (61) |  |  |
| 30 – 45 Min. | 104 (20) |  |  |
| > 45 Min. | 19 (4) |  |  |
| No transportation available | 10 (2) |  |  |

*^1^ Participants with ≤ 50 on WHO Well-Being-Index (WHO-5); ANOVA = Analysis of variances; n = number; SD = standard deviation; dfd = numerator degrees of freedom in the denominator ; dfn = degrees of freedom in the numerator*

**Results of post-hoc analyses**

**General sample**

Table 2 Results of t-tests for dependent samples with Bonferroni correction method to analyse differences of agreement to different implementation options regarding type of consultation at work in a subgroup of employees with depressive symptoms^1^ (n=510)

| **Purpose of consultation** | **Purpose of consultation** | **n1** | **n2** | **Statistic (t)** | **df** | **p** | **p.adj** | **p.adj.signif** |
| --- | --- | --- | --- | --- | --- | --- | --- | --- |
| type_person | type_telephone | 510 | 510 | 30.20 | 509 | <0.001 | <0.001 | * |
| type_person | type_video | 510 | 510 | 25.65 | 509 | <0.001 | <0.001 | **** |
| type_telephone | type_video | 510 | 510 | -4.80 | 509 | <0.001 | <0.001 | **** |

| *^1^Participants with ≤ 50 on WHO Well-Being-Index (WHO-5); df = degree of freedom; *p ≤ .05; **p < .01; ***p < .001; n.s. = not significant (p > .05)* |
| --- |

Table 2 Results of t-tests for dependent samples with Bonferroni correction method to analyse differences of agreement to different implementation options regarding purpose of consultation at work in a subgroup of employees with depressive symptoms^1^ (n=510)

| **Purpose of consultation** | **I Purpose of consultation** | **n1** | **n2** | **Statistic (t)** | **df** | **p** | **p.adj** | **p.adj.signif** |
| --- | --- | --- | --- | --- | --- | --- | --- | --- |
| purpose_occupational | content_maintain | 510 | 510 | -0.60 | 509 | 0.546 | 1 | ns |
| purpose_occupational | content_private | 510 | 510 | 6.65 | 509 | <0.001 | <0.001 | **** |
| purpose_occupational | content_reintegration | 510 | 510 | 2.51 | 509 | 0.012 | 0.073 | ns |
| purpose_maintain | content_private | 510 | 510 | 7.89 | 509 | <0.001 | <0.001 | **** |
| purpose_maintain | content_reintegration | 510 | 510 | 3.39 | 509 | < 0.01 | 0.005 | ** |
| purpose_privat | content_reintegration | 510 | 510 | -4.16 | 509 | <0.001 | <0.001 | **** |

| *^1^ Participants with ≤ 50 on WHO Well-Being-Index (WHO-5); df = degree of freedom; *p ≤ .05; **p < .01; ***p < .001; n.s. = not significant (p > .05)* |
| --- |
